# Pattern recognition receptor mediated downregulation of microRNA‐650 fine‐tunes MxA expression in dendritic cells infected with influenza A virus

**DOI:** 10.1002/eji.201444970

**Published:** 2015-10-30

**Authors:** Tica Pichulik, Elham Khatamzas, Xiao Liu, Oliver Brain, Magno Delmiro Garcia, Alasdair Leslie, Benedicte Danis, Alice Mayer, Dilair Baban, Jiannis Ragoussis, Alexander N. R. Weber, Alison Simmons

**Affiliations:** ^1^ MRC Human Immunology UnitWeatherall Institute of Molecular MedicineJohn Radcliffe HospitalHeadingtonOxfordUK; ^2^ Department of ImmunologyInterfaculty Institute for Cell BiologyUniversity of TübingenTübingenGermany; ^3^ Translational Gastroenterology UnitJohn Radcliffe HospitalHeadingtonOxfordUK; ^4^ Wellcome Trust Centre for Human GeneticsOxfordUK

**Keywords:** Dendritic cells, Host/pathogens interactions, Innate immunity, ISGs, MicroRNA

## Abstract

MicroRNAs are important posttranscriptional regulators of gene expression, which have been shown to fine‐tune innate immune responses downstream of pattern recognition receptor (PRR) signaling. This study identifies miR‐650 as a novel PRR‐responsive microRNA that is downregulated upon stimulation of primary human monocyte‐derived dendritic cells (MDDCs) with a variety of different microbe‐associated molecular patterns. A comprehensive target search combining in silico analysis, transcriptional profiling, and reporter assays reveals that miR‐650 regulates several well‐known interferon‐stimulated genes, including IFIT2 and MXA. In particular, downregulation of miR‐650 in influenza A infected MDDCs enhances the expression of MxA and may therefore contribute to the establishment of an antiviral state. Together these findings reveal a novel link between miR‐650 and the innate immune response in human MDDCs.

## Introduction

MicroRNAs (miRNAs) are small noncoding RNAs, ∼18–22 nucleotides in length, which regulate gene expression on a posttranscriptional level by binding to the 3′ untranslated region (UTR) of target mRNAs, thereby inhibiting translation and/or decreasing mRNA stability 1. Binding of eukaryotic miRNAs to their respective target site is mediated by perfect sequence complementarity between nucleotides 2–8 at the 5′ end of the miRNA—the so‐called “seed region.” miRNA‐mediated gene silencing is central to a range of cellular processes including development, hematopoiesis, oncogenesis, and the immune response 2.

Recent publications have shown that miRNA expression is modulated under a variety of inflammatory conditions and is involved in the dynamic regulation of the innate immune response including its development, maturation, and function 3. Importantly, aberrant expression of selected miRNAs can give rise to impaired innate immune function and has been observed in patients suffering from, for example, Crohn′s disease 4, rheumatoid arthritis 5, 6, or multiple sclerosis 7, 8. Signals initiated by pattern recognition receptors (PRRs), for example Toll‐like receptors (TLRs), upon recognition of microbe‐associated molecular patterns, have been shown to regulate the expression of various miRNAs including miR‐155, miR‐146a, and miR‐21 9. The best‐characterized miRNA in immunity to date is miR‐155, which is robustly induced following PRR sensing, and acts to facilitate induction of the interferon (IFN) response and simultaneously control inflammatory pathways by providing a negative feedback loop 10. However, the full range of PRR‐responsive miRNAs and their role in human antigen‐presenting cells such as dendritic cells (DCs) remains to be defined.

miR‐650 is unique to man and has been previously linked to tumorigenesis. Increased miR‐650 expression levels have been measured in gastric cancer biopsies and it has been shown to directly target and repress the expression of tumor‐suppressor proteins ING4 11 and NDRG2 12. However, challenging its role as a potential “oncomiR,” miR‐650 is induced following overexpression of p16^INK4a^, a tumor suppressor that inhibits cell‐cycle progression 13. In B cells miR‐650 has been linked to immunoglobulin gene rearrangement. It targets proteins regulating cell proliferation and survival including CDK1, ING4, and EBF3 14 and increased levels of miR‐650 were shown to be associated with a favorable prognosis for B‐cell chronic lymphocytic leukemia 14.

In this work, we establish a novel link between miR‐650 and the innate immune response. We show that miR‐650 is downregulated upon PRR signaling in primary human monocyte‐derived dendritic cells (MDDCs) and fine‐tunes the expression of a number of genes with a role in the immune response. In particular, it directly targets interferon‐stimulated genes (ISGs) IFIT2 and MxA via their 3′UTR. Finally, we show that downregulation of miR‐650 enhances the induction of MxA during influenza A infection of MDDCs in vitro.

## Results

### Stimulation of PRRs downregulates miR‐650 expression in human MDDCs

**Figure 1:**
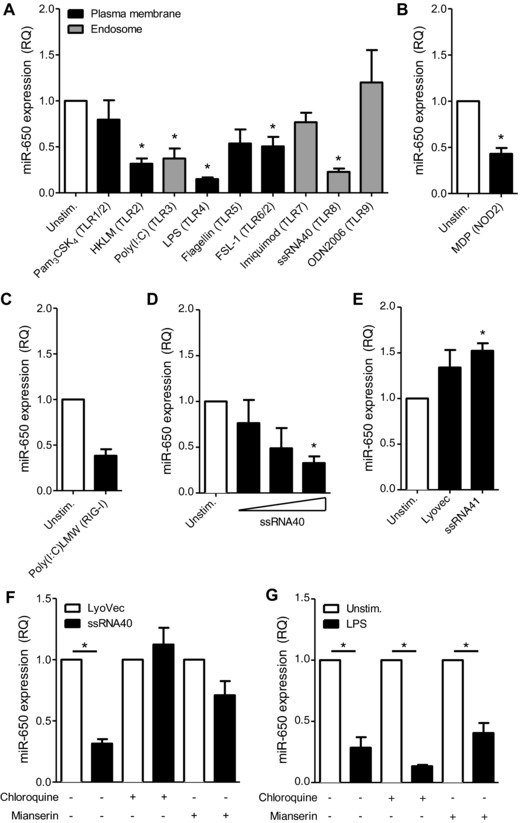
PRR signaling in human MDDCs reduces miR‐650 expression. Human MDDCs were treated with the indicated (A) TLR, (B) NOD‐like receptor or (C) RIG‐like helicase ligands for 24 h. miR‐650 expression was measured by qRT‐PCR. (D) Stimulation of MDDCs with 0.1, 1, or 10 μg/mL ssRNA40 for 24 h. miR‐650 expression was measured by qRT‐PCR. (E) Stimulation of MDDCs with ssRNA41 (10 μg) or LyoVec for 24 h. miR‐650 expression was measured by qRT‐PCR. (F and G) MDDCs were pretreated with 50 μM chloroquine or 30 μg/mL mianserin hydrochloride for 30 min prior to addition of (F) 10 μg/mL ssRNA40 or (G) 1 μg/mL LPS for 24 h. miR‐650 expression was measured by qRT‐PCR. For all figures, miR‐650 expression was normalized against RNU48 and is shown relative to (A–E and G) unstimulated MDDCs or (F) relative to cells incubated with LyoVec. All measurements were performed in triplicate and data are shown as mean ± SEM and are pooled from (A, B, D, and E) four, (F and G) three, or (C) two individual donors. Statistical analysis was performed using a paired Student's *t*‐test (*p < 0.05).

miRNA expression profiling previously performed on human MDDCs in our lab suggested downregulation of miR‐650 occurs upon MDDC stimulation. Further investigation by qRT‐PCR analysis confirmed that miR‐650 expression is downregulated in MDDCs stimulated with a range of different TLR ligands (Fig. 1A), the NOD‐like receptor NLR ligand muramyldipeptide (MDP) (Fig. 1B) or intracellular poly(I:C), which triggers the RIG‐like helicase RIG‐I (Fig. 1C). This effect was independent on the localization of the PRR (plasma membrane, endosome, or intracellular) and the source of the ligand (viral or bacterial). Simultaneous measurement of MDDC maturation markers CD80, CD83, and CD86 by flow cytometry indicated that stimulations leading to full maturation also induced the highest miR‐650 downregulation (data not shown). Stimulation of MDDCs with either imiquimod or CpG DNA failed to alter miR‐650 expression (Fig. 1A). This is in line with evidence that MDDCs lack expression of TLR7 and TLR9 15, 16.

The effect of PRR signaling on miR‐650 expression was further explored by focusing on TLR8‐mediated stimulation by ssRNA40, a short GU‐rich RNA fragment derived from the HIV‐1 LTR 17. Stimulation of MDDCs with ssRNA40 promoted downregulation of miR‐650 with the highest ligand dose showing a significant downregulation (Fig. 1D). In contrast, stimulation of MDDCs with the complexing transfection agent LyoVec or the nonstimulating ssRNA41 failed to reduce miR‐650 levels. In contrary, it led to a mild upregulation of miR‐650 (Fig. 1E). In addition, preincubation of MDDCs with chloroquine or mianserin hydrochloride, which abrogate signaling via endosomal TLRs 18, 19, prevented downregulation of miR‐650 via ssRNA40 (Fig. 1F) but had no effect on TLR4‐dependent regulation (Fig. 1G). This suggests that miR‐650 regulation downstream of TLR8 depends on an intact endosomal signaling compartment.

### Transcriptional profiling reveals differentially regulated genes in MDDCs transfected with miR‐650 pre‐miR

To gain a first insight into the role of miR‐650 in MDDCs, we carried out gene expression profiling of MDDCs transfected with miR‐650 pre‐miR or corresponding scramble control. Transfection of miR‐650 pre‐miR led to differential expression of 67 genes of which six were up‐ and 61 downregulated (fold change >1.2, *p* < 0.01; Table 1). The cutoff was chosen following studies demonstrating that miRNAs can have a relatively subtle effect on the transcript level, which does not fully account for the repression observed at protein level 20, 21. In silico prediction using miRanda and TargetScan identified putative miR‐650 binding sites in five of 61 downregulated and one of six upregulated transcripts. One possible explanation for the discrepancies between in silico prediction and the transcriptional profiling could be that as mentioned previously fine‐tuning of protein expression by miRNAs may be subtle and occur mainly on a protein level. Furthermore, transcriptional profiling also detects secondary effects induced by the miRNA. Lastly, it has been noted before that in silico target searches can produce false‐positive as well as false‐negative results 21, 22, making experimental validation essential.

**Table 1:** Top 20 up‐ and downregulated gene transcripts in MDDCs transfected with miR‐650 pre‐miR

Gene symbol	Gene description	FC
Downregulated transcripts		
CXCL10	Chemokine (C−X−C motif) ligand 10	−1.84
IFIT2	IFN‐induced protein with tetratricopeptide repeats 2	−1.50
GCH1	GTP cyclohydrolase 1 (dopa‐responsive dystonia)	−1.46
CCL8	chemokine (C−C motif) ligand 8	−1.41
FCGR1A|FCGR1B	Fc fragment of IgG, high affinity Ia and Ib, receptor (CD64)	−1.39
BIRC3	Baculoviral IAP repeat‐containing 3	−1.38
PDCD4	Programmed cell death 4 (neoplastic transformation inhibitor)	−1.35
INDO	Indoleamine‐pyrrole 2,3 dioxygenase	−1.35
LTA4H	Leukotriene A4 hydrolase	−1.33
PLXNC1	Plexin C1	−1.32
GBP5	Guanylate‐binding protein 5	−1.32
CCL5	Chemokine (C−C motif) ligand 5	−1.32
COP1	Caspase‐1 dominant‐negative inhibitor pseudo‐ICE	−1.31
RNF170	ring finger protein 170	−1.31
GBP3	Guanylate binding protein 3	−1.30
GBP4	Guanylate binding protein 4	−1.30
OMA1|DAB1	OMA1 homolog, zinc metallopeptidase | disabled homolog 1	−1.29
CASP1	Caspase 1, apoptosis‐related cysteine peptidase	−1.29
SLC39A8	Solute carrier family 39 (zinc transporter), member 8	−1.29
OAT	Ornithine aminotransferase (gyrate atrophy)	−1.29
Upregulated transcripts		
OR52K3P	Olfactory receptor, family 52, subfamily K, member 3	1.33
STAC	SH3 and cysteine‐rich domain	1.26
MOBKL2B	Mps one binder kinase activator‐like 2B (yeast, MOB1)	1.22
RUNX1	Runt‐related transcription factor 1	1.22
C17orf68	Chromosome 17 open reading frame 68	1.21
FAM83G	Family with sequence similarity 83, member G	1.20

MDDCs were transfected with pre‐miR‐650 or scramble control for 24 h and microarray analysis was performed using the Affymetrix GeneChip Human Gene 1.0 ST array. Fold changes (FC) are shown relative to scramble control.

**Figure 2:**
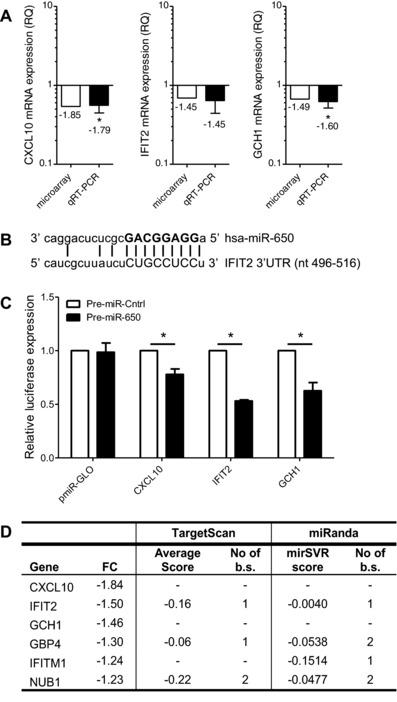
Transfection of MDDCs with miR‐650 pre‐miR in MDDCs reduces mRNA levels of ISGs. Top three transcripts regulated in MDDCs transfected with miR‐650 pre‐miR (see Table 1) were chosen for further validation. (A) Gene expression levels of *CXCL10*, *IFIT2*, and *GCH1* were measured using qRT‐PCR, normalized to GAPDH and expressed relative to scramble control. qRT‐PCR measurements were performed in triplicate and data are shown as mean ± SEM and are pooled from three independent donors. (B) Schematic representation of the miR‐650:IFIT2 duplex with the miRNA seed marked in bold. (C) Dual luciferase reporter assay testing the responsiveness of pmiR‐Glo vectors containing *CXCL10*, *IFIT2*, or *GCH1* 3′UTR sequences to miR‐650 pre‐miR. The empty vector served as negative control. Firefly activity was normalized to Renilla and is expressed relative to control pre‐miRNA. Measurements were performed in triplicates or quadruplicates and data are represented as mean ± SEM from three independent experiments. (D) Table highlighting ISGs that were significantly regulated by miR‐650 pre‐miR on a transcriptional level, including fold changes and in silico target site information (b.s.: binding sites, FC: fold change). (A and C) Statistical significance was tested using a paired Student's *t*‐test (*p < 0.05).

To verify whether the effect of miR‐650 was direct, the three most strongly downregulated transcripts, *CXCL10*, *IFIT2*, and *GCH1* were chosen for further validation. First, the results were validated using qRT‐PCR analysis, which confirms the microarray data (Fig. 2A). Second, in silico target prediction using TargetScan 23 as well as the MiRanda algorithm 24 revealed that the *IFIT2* 3′UTR carries a putative miR‐650‐binding site (Fig. 2B). Therefore, full‐length 3′UTR sequences of *CXCL10, IFIT2*, and *GCH1* were cloned into a dual luciferase reporter construct (pmiR‐GLO) and tested for their responsiveness to miR‐650 (Fig. 2C). As suggested by the target prediction programs, a significant decrease in luciferase activity was observed for the *IFIT2* 3′UTR when cotransfected with miR‐650 pre‐miR. These results suggest that miR‐650 regulates the expression of *IFIT2* by directly targeting its 3′UTR. Surprisingly, the *GCH1* and *CXCL10* 3′UTRs also responded to miR‐650 and may therefore also be direct targets of miR‐650. However, compared with *IFIT2* regulation is more moderate and respective regulatory sites remain undefined.

Interestingly, *CXCL10*, *IFIT2*, and *GCH1* are all ISGs. IFIT2 forms an antiviral complex with IFIT1 and IFIT3 but has also been shown to negatively regulate cytokine secretion in response to LPS activation 25, 26. Antiviral functions have also been demonstrated for GCH1 27 and CXCL10 28. In addition to *CXCL10*, *IFIT2*, and *GCH1* further analysis revealed additional ISGs including *GBP4* 29, *NUB1* 30, and *IFITM1* 31, which were regulated on a moderate level (Fig. 2D). Although the latter three are predicted to contain putative miR‐650 binding sites, they were regulated only very weakly in the microarray. A more detailed study of these targets would be required to determine whether they are in fact direct or indirect targets of miR‐650. Despite the rather mild effect on transcript level, this unbiased approach suggests a potential role for miR‐650 in collectively fine‐tuning a selection of ISGs.

### Screening for miR‐650 targets by combining in silico predictions and luciferase reporter assays

**Figure 3:**
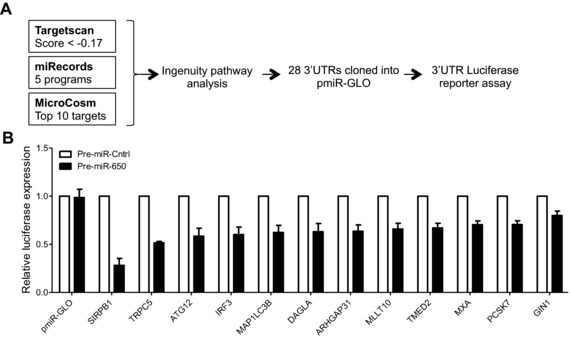
In silico prediction of miR‐650 targets and screening by luciferase reporter assay. (A) Schematic representation of the workflow used to select candidate miR‐650 target genes. A list of candidate targets genes was compiled by considering the top third of all predictions performed by TargetScan (total context score ≤ −0.17) 20, results of the miRecords metasearch as well as the top‐scoring miR‐650 targets retrieved from MicroCosm. Full‐length 3′UTR sequences of 28 putative target genes were cloned into pmiR‐GLO reporter vector and tested for their response to miR‐650 by dual luciferase reporter assay. (B) All pmiR‐GLO constructs significantly (*p* < 0.05) regulated following cotransfection with miR‐650 pre‐miR in dual luciferase screen. pmiR‐GLO empty vector served as negative control. Firefly activity was normalized to Renilla and is expressed relative to control pre‐miRNA. Measurements were performed in triplicates or quadruplicates and data are shown as mean ± SEM and are pooled from three independent experiments. Statistical significance was tested using a paired Student's *t*‐test. All shown targets were significant, *p* < 0.05.

In addition to the microarray approach, we also combined in silico target prediction with luciferase reporter assays to screen for additional miR‐650 targets (Fig. 3A). Three different prediction tools, Targetscan 23, miRecords 32, and MicroCosm 33, were used to generate a list of putative miR‐650 targets. Using Ingenuity pathway analysis the list was narrowed down to genes with a role in the immune response and MDDC function. Ultimately, 3′UTR sequences of 28 candidate genes were tested for their response to miR‐650 by luciferase reporter assays (Supporting Information Table 1). For 12 constructs, luciferase expression was significantly (*p* < 0.5) reduced upon cotransfection of miR‐650 pre‐miR compared with the scramble control (Fig. 3B). In contrast, seven constructs were not significantly regulated upon cotransfection of miR‐650 and therefore considered unresponsive (Supporting Information Fig. 1A). The remaining nine constructs were not significantly regulated; however, luciferase expression was repressed below the level of that of *ING4*, an already validated target that served as a cutoff to define this group (Supporting Information Fig. 1B).

Our screening approach combining microarrays and experimental validation of in silico predictions suggests a number of novel miR‐650 targets involved in important cellular pathways of primary MDDCs, such as innate immune signaling (*IRF3*), autophagy (*ATG12*, *MAP1LC3*), and phagocytosis (*SIRPB1*). More importantly, this approach yielded a number of ISGs, namely *IFIT2*, *GCH1*, *CXCL10*, *IRF3*, *MXA*, and *TRPC5* as novel miR‐650 targets. This provides a new starting point for future work addressing how these target genes are precisely regulated by miR‐650 in a given cell type of interest in different physiological settings.

### miR‐650 directly targets the antiviral ISG MxA and fine‐tunes its expression in MDDCs

**Figure 4:**
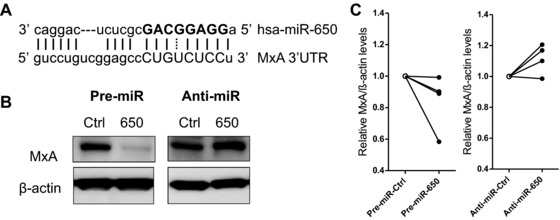
miR‐650 fine‐tunes the expression of the antiviral ISG MxA. (A) Schematic representation of the miR‐650:MxA duplex as predicted by MicroCosm with the miRNA seed highlighted in bold. (B) Effect of miR‐650 on MxA expression levels in MDDCs. MDDCs were transfected with miR‐650 pre‐miR, anti‐miR, or corresponding scramble controls for 24 or 48 h and MxA expression was measured by immunoblot. One representative donor of four is shown. (C) Quantification of immunoblot analysis described above. MxA levels were normalized against β‐actin protein expression and are shown relative to respective scramble control. Data from four individual donors are shown. Statistical significance was tested using a paired Student's *t*‐test and changes in MxA expression were considered to be not significant.

As both target searches revealed an interesting link between miR‐650 and ISGs, we selected one of these targets, the ISG MxA, for further study. MxA is one of the best described ISGs to date. It is an IFN‐induced dynamin‐like GTPase with antiviral activity against a number of different RNA viruses including influenza A virus (IAV) and Thogotovirus 34. Possibly due to the fact that the miR‐650:MxA duplex is characterized by imperfect seed complementarity with a G:U wobble at position 6 of the miRNA (Fig. 4A), only MicroCosm predicts a putative miR‐650‐binding site within the MxA 3′UTR. Due to extensive base pairing at the 3′ end of the miRNA, MicroCosm ranks MxA among the top 50 putative miR‐650 targets and allocates miR‐650 the highest score of all miRNAs predicted to target the MxA 3′UTR. In order to study the effect of miR‐650 on MxA expression, MDDCs were transfected with miR‐650 pre‐miR or anti‐miR and MxA levels were measured by immunoblot. As shown in Figure 4B, transfection of MDDCs with miR‐650 pre‐miR led to a decrease whereas transfection with the anti‐miR resulted in increased MxA expression when compared with their respective scramble controls. Even though quantification showed that this difference was not statistically significant (Fig. 4C), the trend was consistent (Supporting Information Fig. 2). This suggests that miR‐650 may fine‐tune the expression of MxA in MDDCs. It is important to note that MxA is not expressed in unstimulated MDDCs at a steady‐state level. However, we could observe that electroporation itself led to a mild induction of MxA expression in the absence of stimulation (Supporting Information Fig. 2). This may be potentially due to triggering of cytoplasmic RNA sensors or cellular stress and allowed us to detect the effect of miR‐650 on MxA expression in absence of infection.

### miR‐650 fine‐tunes the expression of MxA during Influenza A infection in vitro

**Figure 5:**
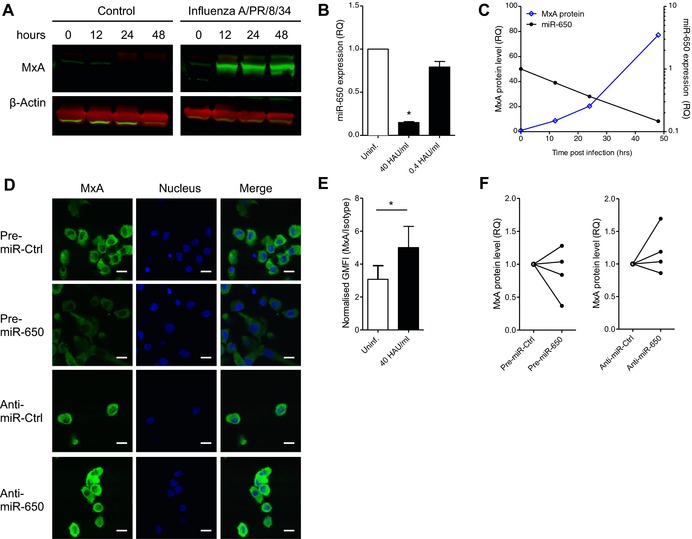
Downregulation of miR‐650 during IAV infection of MDDCs enhances expression of MxA. (A) Measurement of MxA induction during IAV infection of MDDCs. MDDCs were infected with 40 HAU/mL IAV (A/Puerto Rico/8/1934) or left untreated. MxA and β‐actin protein levels were measured at various time points post infection by fluorescent immunoblot (LICOR). (B) Measurement of miR‐650 levels in MDDCs infected with various doses of IAV for 24 h. miR‐650 expression was measured by qRT‐PCR, normalized to RNU48, and shown relative to uninfected control. (C) Comparative time course of miR‐650 expression and MxA protein levels in MDDCs infected with IAV (40 HAU/mL). MxA protein levels were measured by immunoblotting. miR‐650 expression was quantified by qRT‐PCR, normalized to RNU48, and shown relative to infected control cells. (D) Effect of miR‐650 on the expression of MxA in IAV‐infected MDDCs by confocal microscopy. MDDCs were transfected with 83 nM miR‐650 pre‐miR, anti‐miR, or respective controls using Amaxa electroporation. After 24 h cells were infected with IAV (40 HAU/mL) for 6 h and MxA expression was assessed by confocal microscopy; scale bars, 10 μm. (E) Measuring the induction of MxA expression in IAV‐infected MDDCs by flow cytometry. MDDCs were infected with IAV (40 HAU/mL) for 6 h or left uninfected and then stained for MxA or isotype control. The geometric mean fluorescent intensity of MxA was normalized to isotype control. Gating strategy and results from one representative donor of three are shown in Supporting Information Fig. 3. (F) Assessing the effect of miR‐650 on the expression of MxA in IAV‐infected MDDCs by flow cytometry. MDDCs were transfected with 100 nM pre‐miR or anti‐miR using Viromer GREEN. After 48 h MDDCs were infected with IAV (40 HAU/mL) for 6 h and MxA expression was assessed by flow cytometry. Geometric mean fluorescent intensity of MxA was normalized to isotype control. Gating strategy and FACS plots from two representative donors are shown in Supporting Information Fig. 3. (A, C, and D) Data are from one representative donor of two. (B) Measurements were performed in triplicate and results are shown as mean ± SEM and are pooled from two donors. (E) Results are shown as mean ± SD and are pooled from three donors. (F) Results from four individual donors are shown. Statistical analysis was performed using a paired Student's *t*‐test (*p < 0.05).

IAV triggers a multitude of PRRs including TLR3, TLR8, and RIG‐I 35, 36, 37, all of which were found to downregulate miR‐650 upon stimulation (cf. Fig. 1). Although IAV is known to target the IFN response 38, infection of MDDCs with IAV (A/Puerto Rico/8/1934) induced the expression of MxA (Fig. 5A). In addition, IAV infection reduced the levels of miR‐650 with the highest dose showing a significant downregulation (Fig. 5B). Measurement of MxA protein expression and miR‐650 expression levels over time confirmed that these two determinants follow reciprocal expression kinetics (Fig. 5C). This prompted us to investigate the impact of miR‐650 on MxA expression during in vitro IAV infection of MDDCs. Increasing miR‐650 levels prior to infection by transfection of pre‐miR‐650 dampened the induction of MxA, as shown by confocal microscopy (Fig. 5D). Conversely, further reducing miR‐650 levels by transfection of the anti‐miR enhanced the expression of MxA in response to IAV infection. Induction of MxA expression in IAV‐infected MDDCs could also be quantified by flow cytometry (Fig. 5E and Supporting Information Fig. 3B). In order to show the effect of miR‐650 on MxA expression in this experimental system, MDDCs were transfected with miR‐650 pre‐miR or anti‐miR using a lipid‐based transfection reagent, which did not result in the spontaneous induction of MxA expression (data not shown). As shown previously for uninfected DCs, IAV‐infected DCs transfected with miR‐650 pre‐miR also tended to have lower MxA expression whereas DCs transfected with miR‐650 anti‐miR tented to express less MxA relative to scramble control (Fig. 5F and Supporting Information Fig. 3C).

## Discussion

In summary, this study identifies miR‐650 as a novel PRR‐responsive miRNA in a primary human immune cell population. Based on in silico target prediction, transcription profiling and reporter assays, we propose that miR‐650 controls the expression of a number of genes with important implications in the antiviral IFN response. The data indicate that both direct as well as indirect regulation by miR‐650 may be responsible for these effects. One such target with antiviral activity is the ISG MxA, a classical antiviral protein that is known to restrict IAV replication 34. miR‐650 appears to regulate MxA expression in MDDCs by targeting its 3′UTR despite imperfect seed pairing. We show that downregulation of miR‐650 in IAV‐infected MDDCs increases the expression of MxA at the protein level. Additional evidence supporting the finding that miR‐650 expression is reduced following viral infection is provided by a study that found decreased levels of miR‐650 in the urine of patients with hepatocellular carcinoma post‐hepatitis C virus infection as well as hepatitis C virus positive patients relative to the uninfected control group 39. This suggests that miR‐650 may be involved in the antiviral immune response, providing a rationale for the future exploration of the role of miR‐650 in different viral infection settings.

miR‐650 is one of the few miRNAs being downregulated upon activation of primary human MDDCs. Similar to miR‐155 and miR‐146 9, regulation of miR‐650 is neither limited to an individual PRR nor dependent on the origin of the microbe‐associated molecular pattern. Rather our data suggest that the level of miR‐650 regulation inversely correlates with the maturation state of the MDDC. Given that the NF‐κB pathway is a common factor in the regulation of other PRR‐responsive miRNAs, which may be both up‐ and downregulated 9, and due to the observation that miR‐650 is not restricted to a single PRR, we consider it likely that the NF‐κB pathway could play a role in its regulation. Although this will require further in silico and experimental analysis, we speculate whether NF‐κB‐dependent downregulation of miR‐650 may function as a safe guard mechanism that enables the host to boost the expression of ISGs independent from the IFN response, which is frequently blunted by evasion strategies employed by different viruses 40 such as flaviviridae 41. This putative role of miR‐650 in viral infection and evasion strategies warrants further investigation.

Lastly, the role of miR‐650 could be extended to other aspects of the human immune function, as it is expressed in peripheral lymphocytes 42 and, in B cells, its expression is partially coupled to expression of the immunoglobulin λ light chain gene 14. Our main focus was on the role of miR‐650 and ISGs in an infection setting; however, miR‐650 is most prominently known for its role in various types of cancer, including gastric cancer 11, colorectal cancer 12, hepatocellular carcinoma 43, and glioma 44. Many ISGs also have reported roles in cancer; for example, MxA levels have been shown to be reduced in prostate cancer and regulate cell cycle 45. Similarly, GCH1 has been shown to promote tumor growth, angiogenesis, and reduce antitumor immune responses 46. Given that miR‐650 is typically upregulated in cancer cells, we would expect a reduced ISG signature. It could thus be surmised that elevated miR‐650 levels might favor oncogenesis due to reduction of antitumor ISGs.

In summary, this work is the first to show a link between miR‐650 and PRR signaling in primary immune cells and indicates the mIR‐650 may be involved in the antiviral immune response by regulating the expression of ISGs. It therefore lays the groundwork for exploring the role of miR‐650 in different infection settings.

## Material and methods

### Dendritic cell isolation

Buffy coats were obtained from the National Blood Centre (Bristol, UK) following local ethical guidelines granted by Milton Keynes Research Ethics Committee Ref. 07/H0603/43. Monocytes were isolated from Lymphoprep (Axis‐Shield) gradient‐enriched mononuclear cells by MACS CD14^+^ selection (Miltenyi Biotech), and cultured in RPMI 1640 medium (Invitrogen) supplemented with 10% FCS in the presence of 40 ng/mL IL‐4 and 40 ng/mL GM‐CSF (Peprotech) for 5 days prior to use.

### Reagents and antibodies

PRR ligands were obtained from Invivogen and used at the following working concentrations: Pam_3_CSK_4_ (1 μg/mL), HKLM (10^8^ cells/mL), poly(I:C) (25 μg/mL), LPS (1 μg/mL), flagellin (1 μg/mL), FSL‐1 (1 μg/mL), imiquimod (10 μg/mL), ssRNA40 (10 μg/mL), ssRNA41 (10 μg/mL), ODN2006 (3 μM), poly(I:C)/LyoVec (10 μg/mL), and MDP (10 μg/mL). Chloroquine and mianserin hydrochloride were obtained from Sigma. Influenza A/PR/8/34 stock (400 hemagglutination units (HAU)/mL) was kindly provided by Prof. John McCauley (NIMR, London). CD80, CD83, CD86 antibodies for flow cytometry were purchased from eBioscience. Polyclonal rabbit anti‐MxA antibody used for immunoblot and flow cytometry was purchased from Abnova (H00004149‐D01P). The Rabbit (DA1E) mAb IgG XP^®^ antibody (CST, #3900) was used as matching isotype control for flow cytometry. Pre‐miRs and anti‐miRs were obtained from Ambion (pre‐miR negative control #1: AM17110, anti‐miR negative control #1: AM17010, pre‐miR‐650: PM11602, anti‐miR‐650: AM11602).

### Transfection of MDDCs

For microarray analysis, immunoblot and confocal imaging 3 × 10^6^ MDDCs were transfected with 83 nM pre‐ or anti‐miR using the DC Nucleofection kit (Amaxa) according to the manufacturer's instructions (program U‐02). For flow cytometry, 10^6^ MDDCs were transfected with 100 nM pre‐ or anti‐miR in a 12‐well plate using the Viromer GREEN (Lipocalyx). The recommended protocol for suspension cells was used.

### RNA extraction and qRT‐PCR

Total RNA was extracted using the miRNeasy Mini kit (Qiagen) according to manufacturer's instructions. Two‐step real‐time quantitative PCR analysis was performed to assess miRNA levels using Taqman miRNA Reverse Transcription kits and Taqman Universal PCR Master Mix on a 7500 Fast Real‐Time PCR System (ABI). For mRNA quantification, 500 ng RNA were reverse transcribed using the High Capacity cDNA‐to‐RNA kit (ABI). Quantitative PCR was performed with 25 ng template using Taqman Gene Expression Master Mix (ABI). All PCR reactions were set up in triplicate and miRNA expression was calculated using the ^ΔΔ^ *C*t method.

### Microarray analysis

MDDCs were transfected with either pre‐miR‐650 or scramble control at a final concentration of 82.5 nM for 24 h. RNA was extracted using the miRNeasy Mini kit (Qiagen). RNA quality was verified by Bioanalyser 2100 (Agilent). Samples from three separate donors were prepared for hybridization onto Affymetrix GeneChip Human Gene 1.0 ST arrays. Briefly, biotin labeling of cRNA probes and fragmentation were carried out according to standard Affymetrix (www.affymetrix.com) protocols at the High Throughput Genomics Facility (The Wellcome Trust Centre for Human Genetics, University of Oxford, Oxford, UK). Probes (15 μg) were hybridized for 16 h to Affymetrix Human Gene 1.0 ST array whole transcript coverage of approximately 29 000 genes from the human genome by 33 297 probe sets, processed using a GeneChip Fluidics Station 450 according to recommended protocols (EukGE‐WS2v5; Affymetrix). Images were captured using the GeneChip Scanner 3000 (Affymetrix). Transcript levels were determined using GeneChip Operating Software (GCOS1.2; Affymetrix), and data were normalized by global scaling. Robust multiarray average expression was measured by probe sequence information (GCRMA) in BioConductor R statistics and analyzed using Data Mining Tool (DMT 3.1; Affymetrix) and GeneSpring 7.2 (Silicon Genetics); *p*‐values were adjusted for multiple testing using Benjamini and Hochberg's method to control the false discovery rate to determine significantly differentially expressed genes with functional analysis carried out using GeneOntology identifying Gene set enrichments and IPA Ingenuity system used for pathways analysis. Two‐way ANOVA was used to compare the two groups (pre‐mir‐650 and scramble control) and a corrected *p* < 0.01 with an average fold change >1.2 was taken to be significant. All microarray datasets have been uploaded to the Gene Expression Omnibus with the accession number GSE69228.

### miRNA target prediction tools

The following miRNA prediction tools were used for in this study: TargetScan (www.targetscan.org), MiRanda (www.microrna.org), miRecords (http://mirecords.umn.edu/miRecords), and MicroCosm (www.ebi.ac.uk/enright‐srv/microcosm).

### Dual luciferase assay

pmiR‐GLO vector was obtained from Promega and full‐length 3′UTRs were amplified from genomic DNA and inserted using SalI and NheI restriction endonucleases. Primers used to amplify 3′UTR sequences are shown in Supporting Information Table 2. Sequencing was performed to cover the miR‐650‐binding site and the retrieved sequenced are shown in Supporting Information Table 3. HEK293T cells were obtained from CRUK cell bank and grown in DMEM (Invitrogen) supplemented with 10% FCS. Cotransfection of pmiR‐GLO (20 ng) with miRNA pre‐miR‐650 or scramble control (25 nM) was performed in a 96‐well format using Lipofectamine 2000. After 48 h luciferase activity was measured on the Fusion Instrument (Packard) or FLUOStar Optima (BMG Labtech) using the Dual‐Luciferase Reporter Assay System (Promega) according to manufacturer's instructions.

### Immunoblotting

Whole cell lysates were boiled for 10 min in 4× NuPAGE LDS loading buffer (Invitrogen) and cooled on ice. Samples were loaded onto precast 4–12% NuPAGE Bis‐Tris gels and transferred onto PVDF membranes (Hybond‐P, Amersham Biosciences). Blocked membranes were probed using rabbit anti‐MxA (Abnova) or mouse anti‐β‐actin (Sigma) antibodies. For fluorescent detection the blocking step and all antibody incubations were performed in LI‐COR blocking buffer. Membranes were allowed to dry and proteins were detected using the Odyssey Western Blot Scanner (LI‐COR).

### Influenza A infection of MDDCs

A total of 10^6^ MDDCs were resuspended in 100 μL RPMI supplemented with 2 μg trypsin (Sigma) and Influenza A/PR/8/34 was added to cells at the indicated concentrations. After 2 h cells were washed twice with RPMI + trypsin and resuspended in complete medium for further culture.

### Confocal Microscopy

Transfected MDDCs were seeded onto 8‐well poly‐d‐lysine Tissue Culture Slides (BD Biosciences) at 2 × 10^5^ cells/well. Cells were cultured for 2 h to allow for attachment, before infection with IAV (40 HAU/mL) for 6 h. Cells were then washed with 1× PBS, fixed in 4% paraformaldehyde for 10 min at 37°C and permeabilized with 0.1% v/v Triton X‐100 for 7 min. After washing with 2% PBA, cells were incubated with rabbit anti‐MxA antibody (Abnova) overnight and goat anti‐rabbit AF488 (Life Technologies) for 1 h at room temperature. Lastly, nuclei were stained by addition of TO‐PRO‐3 (1:4000, Invitrogen) for 10 min. A Radiance 2000 laser‐scanning confocal microscope was used for confocal microscopy, followed by analysis with LaserSharp 2000 software (Bio‐Rad). Images were acquired in sequential scanning mode.

### Flow cytometry

MDDCs were transfected with pre‐miR‐650, anti‐miR‐650or scramble controls for 48 h prior to infection with IAV (40 HAU/mL) for 6 h. After infection, cells were washed, fixed in 4% PFA for 30 min, and permeabilized by incubating in ice‐cold methanol for 30 min. After washing with 1% PBA, cells were incubated with human AB serum for 5 min at room temperature. Staining with anti‐MxA antibody (Abnova) or matching isotype control (CST) was performed for 1 h at room temperature before addition of the secondary goat anti‐rabbit AF488 antibody (Life Technologies) for 1 h at room temperature. Ten thousand cells were acquired on the FACSDiva Flow Cytometer (BD) and analysis way carried out using FlowJo (version 10). Gating strategy is shown in Supporting Information Fig. 3A.

### Data analysis and statistical testing

Experimental data were analyzed using Microsoft Excel 2007 or Graphpad Prism (version 5). Biological replicates are shown as mean ± SEM and technical replicates are as mean ± SD Statistical analysis was performed using a paired Student's *t*‐test: *p < 0.05. For more detailed information see individual figure legends.

## Conflict of interest

The authors declare no commercial or financial conflict of interest.

**Abbreviations**
HAU: hemagglutination units
IAV: influenza A virus
ISGs: interferon‐stimulated genes
MDDCs: monocyte derived dendritic cells
miRNAs: microRNAs
UTR: untranslated region

## Supporting information

As a service to our authors and readers, this journal provides supporting information supplied by the authors. Such materials are peer reviewed and may be re‐organized for online delivery, but are not copy‐edited or typeset. Technical support issues arising from supporting information (other than missing files) should be addressed to the authors.

Supporting Information
