# Supplementary material for: Pattern recognition receptor mediated downregulation of microRNA‐650 fine‐tunes MxA expression in dendritic cells infected with influenza A virus
Source: Eur J Immunol. 2015 Oct 30;46(1):167–77. doi: 10.1002/eji.201444970 (PMC4738369; doi:10.1002/eji.201444970)
Supplement: Supplementary file 1 — Supporting Information [file EJI-46-167-s001.pdf]

European Journal of Immunology

Supporting Information
for
DOI 10.1002/eji.201444970

Tica Pichulik, Elham Khatamzas, Xiao Liu, Oliver Brain, Magno Delmiro Garcia, Alasdair Leslie, Benedicte Danis, Alice Mayer, Dilair Baban, Jiannis Ragoussis, Alexander N. R. Weber and Alison Simmons

Pattern recognition receptor mediated downregulation of microRNA-650 fine-tunes MxA expression in dendritic cells infected with influenza A virus

© 2015 The Authors. *European Journal of Immunology* published by WILEY-VCH Verlag GmbH & Co. KGaA, Weinheim.

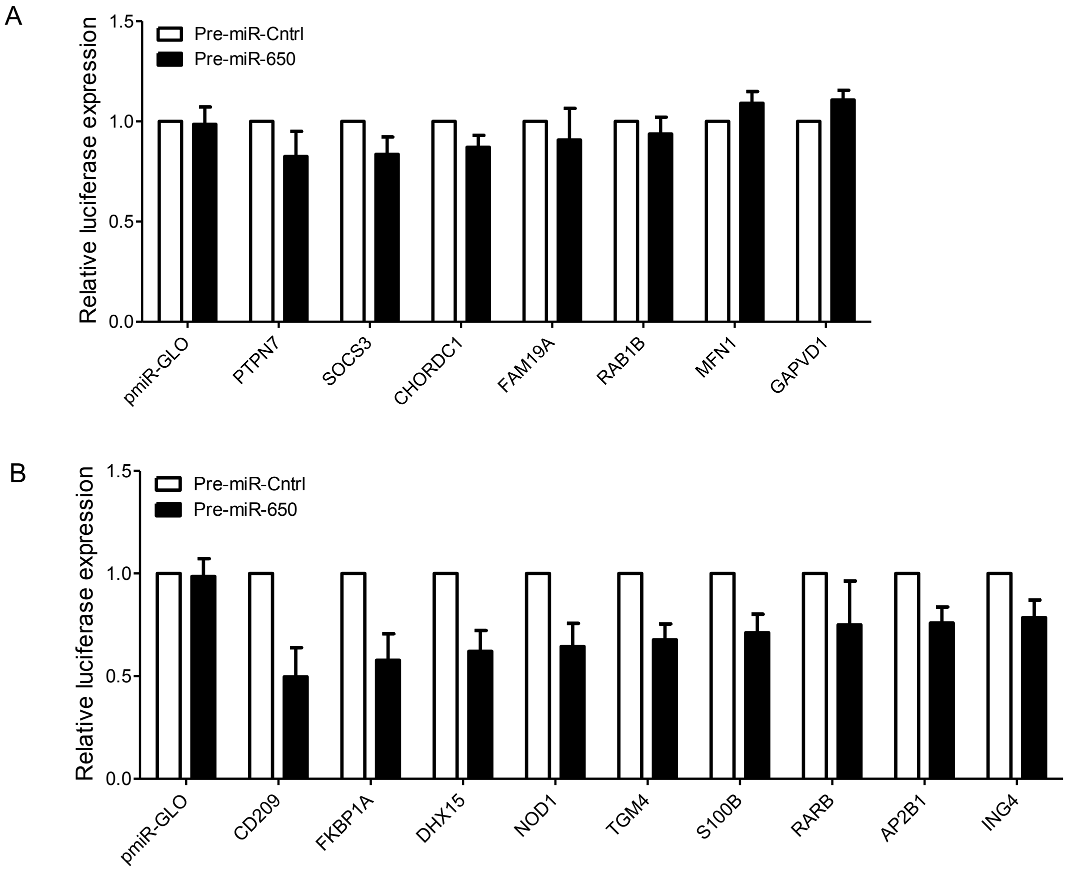

Supporting Information Figure 1 | Results of 3'UTR luciferase reporter assay screen. (A) pmiR-GLO constructs that remained unresponsive to miR-650 pre-miR ($p>0.05$ and regulated less than ING4). **(B)** Genes which were not significantly regulated ($p>0.05$) but relative luciferase expression was repressed below the level of that of ING4, a validated target. **(A, B)** pmiR-GLO empty vector served as negative control. Firefly activity was normalized to Renilla and is expressed relative to control pre-miRNA. Measurements were performed in triplicates or quadruplicates and data is represented as mean ± s.e.m. from three independent experiments. Statistical significance was tested using a paired Student's *t*-test. None of the shown targets were considered significant, $p<0.05$.

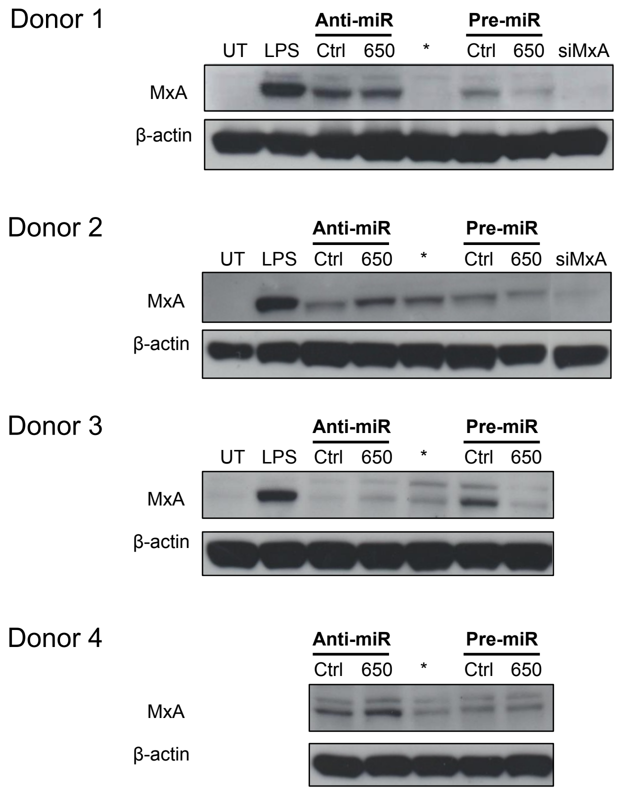

Supporting Information Figure 2 | Effect of miR-650 on MxA expression levels in MDDCs. MDDCs were transfected with miR-650 pre-miR, anti-miR, an siRNA against MxA or corresponding scramble controls using electroporation. After 24 or 48 h MxA expression was measured by immunoblot. UT = untransfected + unstimulated, LPS = untransfected + 24 h LPS. Data from four donors shown.

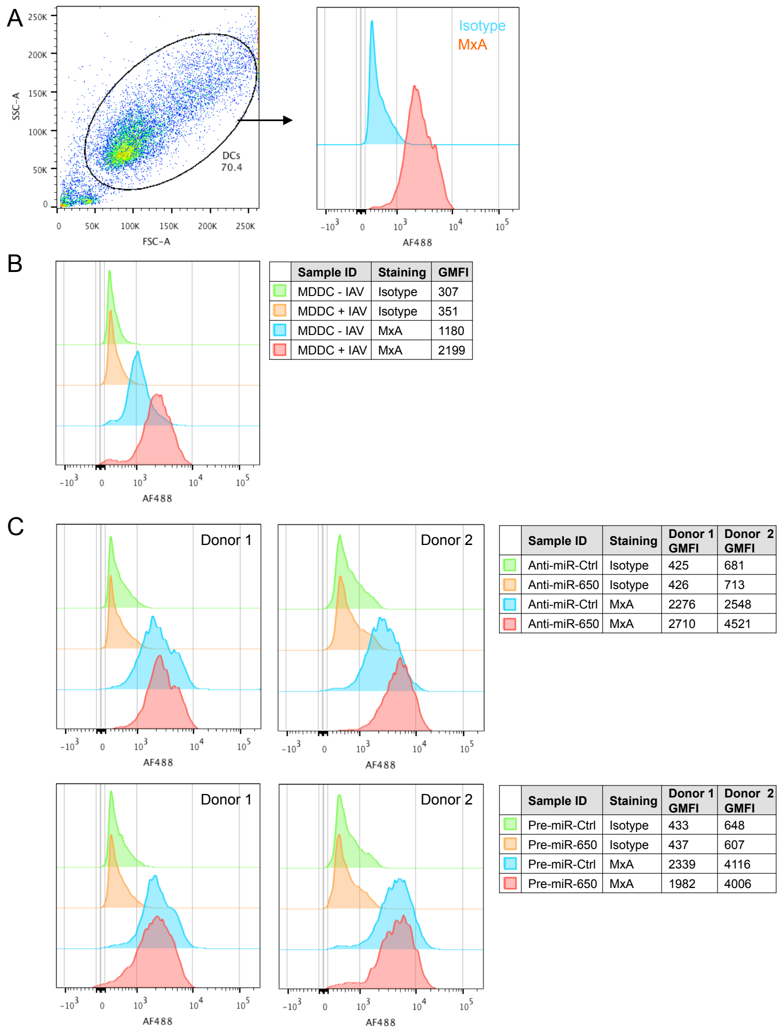

Supporting Information Figure 3 | Quantification of MxA expression by flow cytometry. (A) Definition of MDDCs by FSC and SSC and example of AF488 signal of cells stained with anti-MxA antibody or matching isotype control. **(B)** Induction of MxA expression 6 h post IAV infection (40 HAU/ml). One representative donor out of three shown. **(C)** Effect of miR-650 on MxA in IAV-infected MDDCs. Cells were transfected with pre-miR-650, anti-miR-650 or their respective controls using Viromer GREEN. After 48 h cells were infected with IAV (40 HAU/ml) for 6 h, stained for MxA or matching isotype control and analyzed by flow cytometry. Two representative donors out of four shown.

Supporting Information Table 1 | List of candidates chosen for the 3'UTR screen including *in silico* target site information.

Gene ID	TargetScan		Miranda		MicroCosm	
	No. b.s.	Context score	No. b.s.	mirSVR	No. b.s.	Score
AP2B1	1	-0.27	2	-0.70	NR	NR
ARHGAP31	1	-0.37	2	-0.27	-	-
ATG12	1	-0.34	1	-0.51	-	-
CD209	3	-0.51	6	-0.45	-	-
CHORDC1	1	-0.22	1	-0.59	-	-
DAGLA	2	-0.09	4	-0.46	-	-
DDX17	2	-0.39	2	-0.47	-	-
DHX15	1	-0.23	1	-1.32	-	-
FAM19A	1	-0.40	1	-0.68	-	-
FKBP1A	2	-0.55	3	-0.87	-	-
GAPVD1	1	-0.37	2	-0.48	-	-
GIN1	1	-0.47	1	-0.79	-	-
ING4	1	-0.35	1	-1.03	-	-
IRF3	1	-0.24	1	-0.28	1	18.40
MAP1LC3C	1	-0.24	3	-0.24	-	-
MFN1	1	-0.45	1	-0.97	-	-
MLLT10	1	-0.02	1	-0.34	-	-
MXA	-	-	-	-	1	18.56
NOD1	2	-0.37	2	-0.24	-	-
PCSK7	3	-0.53	3	-0.53	1	18.90
PTPN7	2	-0.53	3	-0.20	-	-
RAB1B	2	-0.05	2	-0.65	-	-
RARB	2	-0.40	2	-1.01	-	-
S100B	2	-0.53	0	-0.00	-	-
SIRPB1	3	-0.63	4	-1.76	-	-
SOCS3	3	-0.04	3	-0.53	-	-
TGM4	1	-0.37	1	-0.97	-	-
TMED2	1	-0.37	1	-0.81	-	-
TRPC5	1	-0.44	1	-1.03	-	-

No of b.s: number of binding sites predicted
NR: gene not found
- : no binding sites found

Supporting Information Table 2 | Primers used for amplification of 3’UTR sequences with restriction sites in capital letters.

Primer	Sequence
CXCL10 FWD	TCGCTAGCaccagaggggagcaaaatcgatgc
CXCL10 REV	AGGTCGACtagtctttcttaatatttccaggagagtacctc
IFIT2 FWD	TCGCTAGCgaatagagatgtggtgcccactagg
IFIT2 REV	AGGTCGACtggaatcagtgacttttatttctcataacagagg
GCH1 FWD	TCGCTAGCcttcattcagtgtgtgtctgcagaacc
GCH1 REV	AGGTCGACaataggaccaacttcaggaacatacatactc
ING4 FWD	TCGCTAGCtaagggccttggattccaacacagtttc
ING4 REV	AGGTCGACgaatgctcagcatgtttactaaaacagtgtc
MAP1LC3C FWD	TCGCTAGCccatgtcgggaaggatgtgtgctc
MAP1LC3C REV	AGGTCGACggacacctgcccacatgcagg
SOCS3 FWD	TCGCTAGCgggtaaagggcgcaaagggc
SOCS3 REV	AGGTCGACgtgctctttattataaattactgaaatgtttcttttctg
CD209 FWD	TCGCTAGCagaacttcaccccccttttaagctacagttc
CD209 REV	AGGTCGACtgcaaggcagccaagcaaggacac
SIRP1B FWD	TCGCTAGCtgaccctcagtctctgctgcctc
SIRP1B REV	AGGTCGACtaatgaagtgacattgagcagagacttgaagg
ARHGAP31 FWD	TCGCTAGCccaagaatggaagcttgtgaggagac
ARHGAP31 REV	AGGTCGACcggagagacaaaacaagaactagagttttaatg
IRF3 FWD	TCGCTAGCccctcgctcctcatggtgtgc
IRF3 REV	AGGTCGACgatcatagcaggaaccagtttattggttgag
RAB1B FWD	TCGCTAGCaggggcacatggagtgggacag
RAB1B REV	AGGTCGACaaaccctcagctcacccgaggg
NOD1 FWD	TCGCTAGCaggatgctttcctgttcatggggtttttgc
NOD1 REV	AGGTCGACaagagtgttttcagtattttattaacaaatgagctggc
AP2B1 FWD	TCGCTAGCaagactggtccagtacccttcaacc
AP2B1 REV	AGGTCGACgtaaatgtgagtttattgacgaagacacaagg
ATG12 FWD	TCGCTAGCccacaaagaaaatcaacttgctactacatgaaatgg
ATG12 REV	AGGTCGACgtgttattccaatatttaggacatagtaggcactc
CHORDC1 FWD	TCGCTAGCtgggagatggaaggaaggctattac
CHORDC1 REV	AGGTCGACttgacaaaccgatagtaaaacatatgctacc
DAGLA FWD	TCGCTAGCaccccagttgcgtggccagc
DAGLA REV	AGGTCGACgtgcttctacatccagtttatacacagctc
MLLT10 FWD	TCGCTAGCaagacttagtgataaaactgggcctgtagc
MLLT10 REV	AGGTCGACacattttgtacattttaatacatgtggaattttacatctagg
TRPC5 FWD	TCGCTAGCtcaagccctaatcatctgttctggaactc
TRPC5 REV	AGGTCGACgtgctgttaatatgagttaggaatgtagccc
FKBP1A FWD	TCGCTAGCaggaatggcctcctcccttagctc
FKBP1A REV	AGGTCGACctgagaaaagccggcataaagcacttttattgc
PTPN7 FWD	TCGCTAGCcccctgccaccctccggtg
PTPN7 REV	AGGTCGACgagtcagtcatccaagaagtctttattttcccac
GIN1 FWD	TCGCTAGCtattcattaaaaacgttgtatagaagaagtatcttgac
GIN1 REV	AGGTCGACggctgaaacaatagaattttattgtctcacag
MFN1 FWD	TCGCTAGCaatagagattgctttggtgaccatgataggagg
MFN1 REV	AGGTCGACgaaactggtgaggcatttccaccagc
S100B FWD	TCGCTAGCattagaaagcagccaaacctttcctgtaacag
S100B REV	AGGTCGACctttttattgaacgcaggccctcgcg
PCSK7 FWD	TCGCTAGCctcagggcctgacagtgtggg
PCSK7 REV	AGGTCGACttggaaggtttttattttggaaaagctgtgcag
PPP2R1B FWD	TCGCTAGCgaggagcaggagggaaaaggcc
PPP2R1B REV	AGGTCGACgaactcatatcagtttaagagaacaactctggag
RARB FWD	TCGCTAGCacattttctagctacttcaaacattccccag
RARB REV	AGGTCGACtatattttattctttgccaggagactccatgg
TMED2 FWD	TCGCTAGCaagcctcttcctgatgatcccaactcag
TMED2 REV	AGGTCGACttaccgaacctggggcatattagatacaacc
DHX15 FWD	TCGCTAGCttcagtgcttagaactgaagttattgagaggac
DHX15 REV	GGTCGACggccaaaattagtataaatagactttattgaagtcagtg
TGM4 FWD	TCGCTAGCcttgtctgatgctgtggagccttagttg
TGM4 REV	GGTCGACagtttaatttaaagcttattgatcatttgagcaaatctctgg
MXA FWD	TCGCTAGCcccacactctgtccagccccgta
MXA REV	AGGTCGACtgtctgctagaaatgagtttatt
EBF3 FWD	TCGCTAGCggacttctgtttaccttccgcagc
EBF3 REV	AGGTCGACcagataaaacaatcttattgtagggtctttaacag
FAM19A FWD	TCGCTAGCccaggagaaatcaagtgatcctcaaggc
FAM19A REV	AGGTCGACatgaaagagagtccattttatatcccaattttattcacc
GAPVD1 FWD	TCGCTAGCcaagaccaaggcccaccaaggc
GAPVD1 REV	AGGTCGACgcaatgtccacatcttcatatttatttccacagtg

Supporting Information Table 3 | Sequencing results of the 28 3'UTR inserts clones into pmiR-GLO vector.

**AP2B1**	AAGACTGGTCCAGTACCCTTCAACCATGCTGTGATCGGTGCAAGTCAAGAACTCTTAACTGGAAGAAAT<br>TGTATTGCTGCGTAGAATCTGAACACACTGAGGCCACCTAGCAAGGTAGTAACTAGTCTAACCTGTGCT<br>AACATTAGGGCACAACCTGTTGGATAGTTTTAGCTTCCTGTGAACATTTGTAACCACTGCTTCAGTCACCT<br>CCCACCTCTTGCCACCTGCTGCTGCTATCTGTCCTTACTTGTGGGCTTCTCCATGCTGTGCCAATGGCTGG<br>CTTTTTCTACACCCTCTTTTGAGTGTAGTTTGGTATTTTGTAATTGAGAGCTCATTTCAAAAGCAGAAAAA<br>GACAACAAATATTAAAGCAAGGAAAAGTGTAACTGAAACACTGCACTTTACTGTTTTATACTTTTGTACA<br>TATGAGAAATCAAGGGATTAGTGCAACCAGTAGAAAGCATTGAAATGACTGTCATTAACCACACAGTCC<br>TGGAGGCAGAGATGCAGTTACCTACCCTAGCTTTTGATGGGTTCTCTTACCTGTAGTAGCCTTATCCCTG<br>GTCATTTGGATTTTCAGTTTGCTTTTTTCTTTTTTTCCCCTCCAAACTCCTTTTCCTTGGCCAAGCCTTCATG<br>CTTCCCCCTTTCCATATTATAATCTCATTTGATTGCTCTGCAGTTGGGAACGGTGATCTTCTTGAATGATG<br>TTTCAGTGTGC[...]CCTTCTTGGGACTTATTCCTGGAGTCAGTGGATACAAGTAGTGCAGAAGGTTCACA<br>CTGCAAATAGTGTTCTCATCTCAAAGCAAACTATCATTCCAGAAGGAAAAGTGTGTCAGGGCAAGCAGA<br>CAACACAATTTCCTATCAGAATATGTCCCTCAACCCCCGAAACAAGGCTTCTCTCAGCCTCCCCACCAGTG<br>ATGGATAACAGCTCCTATTCTCAGCTGACCTGACTGAGCCAACCCATGAACTCTTCACTCCTTGGGGAAG<br>CCACCTCCCATCACACCCCTGAGCAGAGTTAGGGAGGAATTCTACTTCCCATAAAAGGACCTCTCCTGAG<br>AGGCAAAACCTGTTGCCTCCACCACGGCTTCCCTCTTGGCTCATTCCAAGCTTGGCCAAATTGGGGAAGT<br>GGGATGGAGGTTGCCCTGCATCCCCCCTCCTCTGCCTGAGTGTGTCTTTGTAATGTCAGCTGGCATCATA<br>CAAAGAGCAGGAGAAGCAAACACCCAGAACTCTTTTGCTGGTCAGAGATTCCCTGAGTGTCTGTCCTCA<br>CCCAAGCCTGCTCTGTGTCTGTGTTGTGAAGCTTGAGACTCTGGAAAGAAATGGGGAGGGGGGGCAGG<br>GGAAATGTTGCCCTAAGAATGCTTCTCATTCCTCTGTTCTTATTGGGTCCTGTTTTTCGGGAGGGTGGGG<br>GTTGGGGGAAGCTTGACCTTGTGTCTTCGTCAATAAACTCACATTTAC
**ARHGAP31**	CCAAGAATGGAAGCTTGTGAGGGTAAACTGTGGCTTATTCTTACAAAAAGTGTAATAAAGGAGACTGAC<br>CCCTGACAACATGGTAGGCACTGTATACATACTTGTTTTTGTTTTTTGTTTTTTAGAGACGGACTCTATGT<br>TGCCCAGGCTGTTATGGAACTCCTGAGTCAAGTGATCCTCCCACCTTGGCCTCTGAAGGTGCGAGGATT<br>ATAGGCGTCACCTACCACATCCAGCCTACACGTATTTGTTAATATCTAACATAGGACTAACCAGCCACTG<br>CCCTCTCTTAGGCCCCTCATTTAAAAACGGTTATACTATAAAATCTGCTTTTCACACTGGGTGATAATAAC<br>TTGGACAAATTCTATGTGTATTTTGTTCTGTTTTGCTTTGCTTTGTTTTGAGACGGAGTCTCGCTCTGTCAT<br>CCAGGCTGGAGTGCAGTGGCATGATCTCGGCTCACTGCAACCCCCATCTCCCAGGTTCAAGCGATTCTCC<br>TGCCTCCTCCTGAGTAGCTGGGACTACAGGTGCTCACCACCACACCCAGCTAATTTTTTGTATTTTTAGTA<br>GAGACGGGGTTTCACCATGTTGACCAGGCTGGTCTCGAACTCCTGACCTGGTGATCTGCCCACCCAGGC<br>CTCCCAAAGTGCTGGGATTAAAGGTGTGAGCCACCATGCCTGGCCCTATGTGTGTTTTTTAACTACTAAA<br>AATTATTTTTGTAATGATTGAGTCTTCTTTATGGAAACAACTGGCCTCAGCCCTTGCGCCCTTACTGTGAT<br>TCCTGGCTTCATTTTTTGCTGATGGTTCCCCCTCGTCCCAAATCTCTCTCCCAGTACACCAGTTGTTCCTCC<br>CCCACCTCAGCCCTCTCCTGCATCCTCCTGTACCCGCAACGAAGGCCTGGGCTTTCCCACCCTCCCTCCTT<br>AGCAGGTGCCGTGCTGGGACACCATACGGGTTGGTTTCACCTCCTCAGTCCCTTGCCTACCCCAGTGAG<br>AGTCTGATCTTGTTTTTATTGTTATTGCTTTTATTATTATTGCTTTTATTATCATTAAAACTCTAGTTCTTGT<br>TTTGTCTCTCCG
**ATG12**	CCACAAAGAAAATCAACTTGCTACTACATGAAATGGATTTTCACGGAAGAGACAGCTCTGAAAAGTTTT<br>GATGCTTGTGGCAAGAGACTTAACAGATGTGATCTATTTAGTATGTGTCTACTCTATGTTTATGCATAAG<br>AAAACATCCATAGCATGAATGGACTCAGAAAAATGTGATTTGTATTAATGCACCAGTCATCATAAAAGA<br>TGGTCATGATAGTACACCCATTGCTCCTACTTGTTACTATTATTGCTGCAGATCTGCCTCCAAGGTTGAAA<br>AGGAGACTAAGACTGTATAAACATCTTCATTGTCAGTTCTCAAAATGACTGAAATTGTTTTCATGGTAAA<br>AGTTAATATACTAAAGGGTTCCTTTTTTTTTAATGTTTACATTTATCTCTATGTTTACCTTTTTAGTCACATT<br>GACCTGCTGGCTGAATACCTCAAATAGTCCAGTAGAGGGCAGTCCACCAGGCAGAAAAGGTTAGGCGT<br>TTTGGTTTCACATCTTTGCTGGGGAATAATAGGGGAAATGGCTGTTTTTGCTAATTTTTAGCTAATATCTA<br>GCCAGGAGAGCAAGCACATAGGACAGACTGAAAGACTGTAATTTTACACAATACACATGGCTTAATTAT<br>TTTATTGGGATACAGAAAAATATAAATTCTGGACAAATAAGTCATATACCTGTTTTCAGTCCTAACATTTA<br>AGGATTCTTGAGTCCCAATCACATAACTGTGGTGTTACTCTGTCATTTATATGGTGTCAAAAGCACTTGA<br>TGAGTAAACCCA[...]TGATTCAGATTTTAACTTTTTAAAGGCTATCTGAATCATTCAAGGAGAAGATACCC<br>TTTCTCTCATAAAAACACTTAGAGCAAACTACCACTATTAAATCACTTATTGCATACTGCCTCAGAGCCAT<br>GTGTCCAGTTATTCTGGTTTTTATCTGGTACCCACACACACATAATACTTAGTACATTTCCTTATATGTAAA<br>TAATTAGTAATGTAACACATCGAGTGAATGAAATTGTGATTAGCAAAGTATGACAGCTAAAAACAAAGG<br>AAATTTTGTATAGTTTAAGTTTTTGTACAGCAGAAGGTAATGGAAAATATTTGCAACATATGACAGTGTT<br>TTTAATGTCTTAAGTATATGAAGAATTTGTTTCAATTGGTAAGAAAAGACCAACTGAAAAATAGGCAAA<br>ACATACCAAATTTCGATTCACAAAAAGATACAAATGGCCAACAAAACATGAAACACCTTGAACCCCATTG<br>GTTACAAAAAATACAAATAAAAACAATGCTGACTGACAATAAAAATAATGTACATCTTGCTATTAGCAGA<br>GTTGTGGGGAAATCCTGTCATATTTTGGTGAATAGAATATGAGTTGGTATATCACTTTTAGGGGGCCATT<br>TGGCAATATCAAAAACCTTGAAACGTAAGTATAAACTTCAGTTTGTTCGTATAACCTGTCATTCACATATT

	GAGTGCCTACTATGTCCTAAATATTGGAATAACAC
**CD209**	CAGAACTTCACCCCCTTTTAAGCTACAGTTCCTTCTCTCCATCCTTCGACCTTCACAAAATCTCTGGGACTG TTCTTTGTCAGATTCTTCCTCCTTTAGAAGGCTGGGTCCCATTCTGTCCTTCTTGTCATGCCTCCAATTTCC CCTGGTGTAGAGCTTGTTTTTCTGGCCCATCCTTGGAGCTTTATGAGTGAGCTGGTGTGGGATGCCTTTG GGGGTGGACTTGTGTTCCAAGAATCCACTCTCTCTTCCTTTTGGAGATTAGGATATTTGGGTTGCCATGT GTAGCTGCTATGTCCCCTGGGGCGTTATCTCATACATGCAAACCTACCATCTGTTCAACTTCCACCTACCA CCTCCTGCACCCCTTTGATCGGGGACTTACTGGTTGCAAGAGCTCATTTTGCAGGCTGGAAGCACCAGG GAATTAATTCCCCCAGTCTACCAATGGCACCCAGAGAGGGCATGGAGGCTCCACGCAACCCCTTCCACC CCCACATCTTCCTTTGTCTTATACATGGCTTCCATTTGGCTGTTTCTAAGTTGTATTCTTTATTTTATTATTA TTATTACTATTTTTCGAGATGGAGTTTCACTCTTGTCGCTCAGGCTGGAGTGCCATGGCGCGATCTTGGC TCACTGCAACCTCTGCCTCCCGGGTTCAAGTGATTCTC[...]TGATCCAGCATCTTCTGGACAGCTTCCGGT TTTTGTTTCTTCTGTCGTTTCTGTTCCTCCTCCTCTCTCTCTTTCCTCTGCTGTTCTTCCCATTGTTCCTTTAA CTTTCGCTCTTGTTCTTGCCGTTTTCTAGCCACCTCTTCCTTTTCCTTCTTTATTCTGAATTCTTCTTGTGCCT TCTGCTCTCTCAGCAACCACTCCTCATGTAATCTTTGCCTCTCTCTTCCCCATAGCTTTTCTAGTTGTTGTTT TTCAATAAAAGTGTCCTCCTCTTTCTGTGAGAGTCCTGAGTCCCTCAGTGGAGCAAGTTCCTGCTGGCGT TTCTTTCGTTTCTCCTTCTTCAGGGCGGCCCTGTACTTTTTGTGGCTTGGTTTCTCTGGAAATGTCACCTTT TCGGGCGCAGCCATCTTGCCGGCACCGCCCCGCCCCTCTAGTTGTATCCTTTATAATAAACTGGTAAACA TTGTAACCGCAGATTCAGCCCAATCTGGTTCAACTTTGTGTAATAAAATGGCGAGTTGTTTTTCAGTTGTC GTGGACCCCCAGGTTGCAAGTTACATACCCTGGGCATGTCCAGATGAACGAAGCGTGCAAATCCACGTG GAACCTAAGTGCTCAGACCGAGGAACAGGGACTGAGTTAAGAAGTGGACACCACGTGGCATGATCCTT GATCCAATCAGATTGAGCCCTGGCGTGATCCAGTCAGATCAAGCCTCCTGAATCCCCTCATTACAAGATC CAATCATATCATGCCTCACTACCCTCTGTATATAAAATCTGCCCCAGCCTCCAACTTGGAGAGACAGATTT GGGCCAGACTCCTGTGTCCTTGCTTGGCTGCCTTGCA
**CHORDC1**	GKRAAGGGCGCAAAGGGCATGGGTCGGGAGAGGGGACGCAGGCCCCTCTCCTCCGTGGCACATGGCA CAAGCACAAGAAGCCAACCAGGAGAGAGTCCTGTAGCTCTGGGGGGAAAGAGGGCGGACAGGCCCCT CCCTCTGCCCTCTCCCTGCAGAATGTGGCAGGCGGACCTGGAATGTGTTGGAGGGAAGGGGGAGTACC ACCTGAGTCTCCAGCTTCTCCGGAGGAGCCAGCTGTCCTGGTGGGACGATAGCAACCACAAGTGGATTC TCCTTCAATTCCTCAGCTTCCCCTCTGCCTCCAAACAGGGGACACTTCGGGAATGCTGAACTAATGAGAA CTGCCAGGGAATCTTCAAACTTTCCAACGGAACTTGTTTGCTCTTTGATTTGGTTTAAACCTGAGCTGGTT GTGGAGCCTGGGAAAGGTGGAAGAGAGAGAGGTCCTGAGGGCCCCAGGGCTGCGGGCTGGCGAAGG AAATGGTCACACCCCCCGCCCACCCCAGGCGAGGATCCTGGTGACATGCTCCTCTCCCTGGCTCCGGGG AGAAGGGCTTGGGGTGACCTGAAGGGAACCATCCTGGTGCCCCACATCCTCTCCTCCGGGACAGTCACC GAAAACACAGGTTCCAAAGTCTACCTGGTGCCTGAGAGCCCAGGGCCCTTCCTCCGTTTTAAGGGGGAA GCAACATTTGGAGGGGACGGATGGGCTGGTCAGCTGGTCTCCTTTTCCTACTCATACTATACCTTCCTGT ACCTGGGTGGATGGAGCGGGAGGATGGAGGAGACGGGACATCTTTCACCTCAGGCTCCTGGTAGAGA AGACAGGGGATTCTACTCTGTGCCTCCTGACTATGTCTGGCTAAGAGATTCGCCTTAAATGCTCCCTGTC CCATGGAGAGGGACCCAGCATAGGAAAGCCACATACTCAGCCTGGATGGGTGGAGAGGCTGAGGGAC TCACTGGAGGGCACCAAGCCAGCCCACAGCCAGGGAAGTGGGGAGGGGGGGCGGAAACCCATGCCTC CCAGCTGAGCACTGGGAATGTCAGCCCAGTAAGTATTGGCCAGTCAGGCGCCTCGTGGTCAGAGCAGA GCCACCAGGTCCCACTGCCCCGAGCCCTGCACAGCCCTCCCTCCTGCCTGGGTGGGGGAGGCTGGAGG TCATTGGAGAGGCTGGACTGCTGCCACCCCGGGTGCTCCCGCTCTGCCATAGCACTGATCAGTGACAAT TTACAGGAATGTAGCAGCGATGGAATTACCTGGAACAGTTTTTTGTTTTTGTTTTTGTTTTTGTTTTTGTG GGGGGGGGCAACTAAACAAACACAAAGTATTCTGTGTCAGGTATTGGGCTGGACAGGGCAGTTGTGTG TTGGGGTGGTTTTTTTCTCTATTTTTTTGTTTGTTTCTTGTTTTTTAATAATGTTTACAATCTGCCTCAATCA CTCTGTCTTTTATAAAGATTCCACCTCCAGTCCTCTCTCCTCCCCCCTACTCAGGCCCTTGAGGCTATTAGG AGATGCTTGAAGAACTCAACAAAATCCCAATCCAAGTCAAACTTTGCACATATTTATATTTATAT
**CXCL10**	ACCAGAGGGGAGCAAAATCGATGCAGTGCTTCCAAGGATGGACCACACAGAGGCTGCCTCTCCCATCA CTTCCCTACATGGAGTATATGTCAAGCCATAATTGTTCTTAGTTTGCAGTTACACTAAAAGGTGACCAAT GATGGTCACCAAATCAGCTGCTACTACTCCTGTAGGAAGGTTAATGTTCATCATCCTAAGCTATTCAGTA ATAACTCTACCCTGGCACTATAATGTAAGCTCTACTGAGGTGCTATGTTCTTAGTGGATGTTCTGACCCT GCTTCAAATATTTCCCTCACCTTTCCCATCTTCCAAGGGTACTAAGGAATCTTTCTGCTTTGGGGTTTATCA GAATTCTCAGAATCTCAAATAACTAAAAGGTATGCAATCAAATCTGCTTTTTAAAGAATGCTCTTTACTTC ATGGACTTCCACTGCCATCCTCCCAAGGGGCCCAAATTCTTTCAGTGGCTACCTACATACAATTCCAAAC ACATACAGGAAGGTAGAAATATCTGAAAATGTATGTGTAAGTATTCTTATTTAATGAAAGACTGTACAA AGTAGAAGTCTTAGATGTATATATTTCCTATATTGTTTTCAGTGTACATGGAATAACATGTAATTAAGTAC TATGTATCAATGAGTAACAGGAAAATTTTAAAAATACAGATAGATATATGCTCTGCATGTTACATAAGAT AAATGTGCTGAATGGTTTTCAAAATAAAAATGAGGTACTCTCCTGGAAATATTAAGAAAGACTA
**DAGLA**	ACCCCAGTTGCGTGGCCAGCCGGGCCCAGGCAGGAGCAGGTGGCCCTGTGGGCACCTGGTGCCTGCCC CCTGCCGGGCAGCTTTAAGGACAGACCCCCAGGGGCAGTTTAGCCTCAGGCACAGGCATCGCTGCTGA GCTGGGGGTCCGCATCCCTACCTCAGCTTAGGACCCCCAGAGCCAAGGTGGCTGGGATCTGGCCCCACA GATGGGGAAAGATGGGGAAGGGTGTGGAGTGGGGAGGAGCCTGGGCAGCCTGCTGGGTGGGCCACA CTCAGCCTGACTGCCCTCCATGGGGGCATTCTGGCACCCCCTGCTCCAGGACAGGCCATGGGCAAGCTG

	CCTCCCATCACTGCCTGCTGGCTGCTCTCCCAGGGGCCAGGTGGAGAGCAGTGCCCCCCGACACATGTA TTCTCATCTGTGGTCCAGGCCGGCATCGTCCTGGCCACCCCCCAGATCTGGTGCCTGCTGGCCGGCCCCC TGGGGTGCCCCTGCCGAGGTGGCCTGCAGTGCTGTACATGTTTACAGAAGCTGCTGGGCTTGGCTCAG GATGTGTTCTGGGCTTGCAAGCCCCCCGCCCAATCATGTGTTCAGTAGCCGTCCTCTGAGCAGGGCCCA AGGCAGCCAGGGGCCTGGAGGGGCCAGAGGAGGGTGGGGTCAGGGCCGCCCCTTCTCTGCCTTGTGC CTCTCATGCTGCCTCCTCTGCCCATGGGTCCTGGGCACCCAGGCCTGCCCTGCCTGCTGGCTACTTCCTG GCTTACCTTCTACCCCCAAGGATCCTCACCACCCAAAGGGTGGTGGGCACTGCTGTGACCACCCCAGCTG CAGAGTCAG[...]CCCTTGAAGGATGGGCCTGCTGCACGTCTCCCTCCTCCACCCCATACCACACTGGGGG GTCTGAGCCACCCCCCTCAGCCCCGTTCGGCTCAGACCGACCCCCACTCCATCCCCAGACCTGCAGCACA AGTGCGCGGGCCTGTCCTCCCAGGGGCCTGGGCGACTCCATATGCAATCAGTAGCGAGCAGCCGGGCC CCACAGACCCTCATGCACTCTCTTACGTGCCATTCTCCCCAGACTTTTTTTGTACTTAATGTATGAAAGAT CCAAACTAATATTGCTGTAAAAAGGAGAGACAAATTAATATAGCTTATTCTATAAATATATCTGTATATA AAGGTTTCTGTATATTGTATAGAGCTGTGTATAAACTGGATGTAGAAGCAC
**DHX15**	TTCAGTGCTTAGAACTGAAGTTATTGAGAGGACAGCTTTAAAAGATGAATGAACTCAAAAGTTCGAGTT GTGCTCTTCACGTTGGTTCGATAATGGCCTTTATTTGAAAGCTTTTTAATTTTTCTTTACAGTAAATATTCC ATTCTGATTTCATAAATTAAACATTTATGCCTCCCTTTTGTGTTGACACTGTAGCTCATACTGGAAAAGTC GATCAATGTTTTGCAGTTTATTGAAAGTAGTTCTATATATAACAATGTTATAAGCATTTCTTTAGAAATGG TTGAAAATGCTTCTAAAATGTGATTATCGACCATGGTATGCATGATCGTTGTAATTGTTGACATTCCTTTT AGAAGTTGTGAAATGTTACAACTTGTGCTTATGTAGACACAATCTTCTGTCTCAGTACAGAGGCACTGAC TTCAATAAAGTCTATTTATACTAATTTTGGCC
**FAM19A**	CCAGGAGAAATCAAGTGATCCTCAAGGCTGATGACATTGAACATGCGCATAGAAACTTAACTCAACTCC TGAGGTGATCTTGAAGATTTTTATACCACTTGAAAGAGGCGCTCAATAGTCTATTTCCAAGGGATTTCAT GGCCTCTTCTTGAAATCAAGACTTTTTAAAAGTCAGACATGAACTTGCATGTCATGAAGATTTCAGCAGA TTTGAACTGTGTTCAACTTGTAAATTGTTAAAAGAATTTGAAGTCACTGTCTGAGGAGCTGGTGAAGAG TTGTTTTTTTCAGGGTGATGTTAGAGACAGTCACCTTTTGAGTTATTGGCTCCAGATGTGACTACTTTTCT TGTTTCTGCAAGCTGTATCCCAAGTGCACTGTCCTTCTGTCCTGGATGTGTTCCTGGGTCCTATGTTCATT TGCTAGTGGGACTACACATGGCTTTAATGACATTTCCTTTGAGAACTTTTCCTCTGGCATGGTGTAGACT GAGACAATTTTATTTATATCCTAATCTTGGAGCTCAGAAAGCCTACATGTTTTAACATCTTAAAGTTGCTT TTGTTAAAGGAATGGAAATATATATCCATTGGTAATAATGTTGGCAAGTAATAGTTATCTGAATAAATCA ATCATATAAGAATGTATAGACAAGCTGACATATTTCCCTAAGGCTAACAACACCCTGCTGAAGCTCTTTG TCAAATAGGTAGTAGTTAGAACTGGATTGCCATTTTCATTATATAATACTTGTACCTCTAGAGCACTCTC CCTTTCTG[...]GAGAGAAGAAAGAAAAGTGCATAGCCATTCTGTAACAATATTGTGTAAACCTATAGTTT GAAGGAATGCAAGGAGAAGGATTTCTGTGTTTTACTCATTTTAGGCTGTTCAGAAGATGCTTCAAAAATT GTCCTGTTAGAATTTCCATCATGGAAGGTGGTATGGAAGAAGGTATGGAAATACTTTGTATTCTAAAAA CTCACTGACGTGGTCAGTTAGACATACGTTGGTTTCCAGGATGGAGGCCCATATATCCTGGGGAGCTTT GGTCTATTAGTTTGTGACAATATTCAAAGGCCAAAACACTACTCAGACACTTTCCTGGGAAGAGCAACTA AAAATGTAAAATTGGTTAAAAATAAAATCTGAAAAGTATGTATCTCACATTGAACTAAAATCCACTGTCT CATAAGTTCATGGAATGAAATGGCTTTCTGCCTCCATTTTAATCATGCATAAAATGAATTAGATGGCTTT GAGTGGATTTTCACAATGGCTCAAGACTATATGAAATTATAAAAAAAAAGTTGCCCTGGGGTTTCTGCAT CAATTAGAATATCATTAATTTCTTTGTAACCAAGTGAAAAACTATACTTTTTGGAAATTATGAATTTGTCC TAGGTTTGTTTGAGATTTGAAATTATACATCATGCTTCTCATTTTTTAAACTATGTTCTTTAAATCAACACT GGAAACTCTGTATTATATACAAGTGTAATACATGCATATAATAGAAAAAAAACATGGAATTTCAAATATA CTAACTAGATTATCCCCAGTAGATTAATGTTGTGACTATTCAGAAAAGGTGAATAAAATTGGGATATAAA ATGGACTCTCTTTCAT
**FKBP1A**	CAGGAATGGCCTCCTCCCTTAGCTCCCTGTTCTTGGATCTGCCATGGAGGGATCTGGTGCCTCCAGACGT GTGCACATAAATCCATATGGAGCTTTTCCTGATGTTCCACTCCACTTTGTATAGACATCTGCCCCAACTGA ATGTGTTCTGTCACTCAGCTTTGCTTCCGACACCTCTGTTTCCTCTTCCCCTTTCTCCTGGTATGTGCGTTT ACCTAAACTATATGCCATAAACGTCAAGTTATTCATTTTACTTTTTCATTTTGGGGTGAAGATTCAGTTTC AGTCTTTTGAATATAGGTTTCCAATTAAGTACATGGTCAAGTGTTAACAGCACAAGTGATAAGTTAACGT TAGGATAGGAATTGGTGTTGAGGGGGGTTGCAAGAATATTTTATTTTAATTTTTTGGATGAAATTTTTATC TATTATATATTAAACATTCTTGCTGCTGCGCTGCAAAACCATAGCAGATTTGAGGCACTGTTGAGGACTG AATTATTCTCCAAGTTGAGAGATGTCCTTGGGTTAAATTAAAAGCCCTACCTAAAACTGAGGTGGGGAT GGGGGAGAGCCTTTGCCTCCA[...]TCAATTGAAGGTGCTGTTTGTAGACTTAACACCCAGTGAAAGCCCA GCCATCATGACAAATCCTTGAATGTTCTCTTAAGAAAATGATGCTGGTCATCCCAGCTTCAGCATCTCCTG TTTTTTGATGCTTGGCTCCCTCTGCTGATCTCAGAGTTTCCTGGCTTTTCCTCCCTCAGCCACTTCTCACCC CTTTGCTGTCCTGTGTAGTGATTTGGTGAGAGGAATCGTTGCTGCCCCCTTCCCCTAGCACCATATATGA GTCTCAAGTTTTATTATTGCAATAAAAGTGCTTTATGCCGGCTTTTCTCAG
**GAPVD1**	CAAGACCAAGGCCCACCAAGGCAGCAGACTGTTAATCAGACAAACAGATCTCTGAGAAGGTGCATCAG CTGCTTTGAAGGCTGAAGATTGTTTTGTATGATACTGCACAGCATCAGGCATTTTAAAGCAGATCTTTAC TAAACAGGTTAATGAGCTAACAAGCAGGTTCTCTCGTCTTTGGGCTCTTTCCTTTCTGAGTTGCATATTCT ATTTTCTTGTCCCCAAGTAGAGACTAGTACTACAAAAAGGGACCACATTTTTCAAGTATTTCTAAGTATAA AAAACAAAACAAAAATCTCTTAGGAAATGTCTAGACCTCCATTCTTGGATTCCCTTTCTTTCCTTTTATTTT AAAAAAGAACAGTACCCCTCTTTTAAGATGCTGTCTTACATTAATGAGCATCTAATGGAAAGAAGGTAT

	GAGTTGCACTGAGGATTAGAATAGTGGTGCGTTAGTGGCATTATCTATAAATACACTCACCTAAATTGA<br>AAGCTAAGAAGGAAATGTAAATATAATATATATTTATATTTGATGTAATATGGACATCTGCAGATTCTAA<br>TAAACAAGGACTATTGCTGATAGTAGGCTGTGACATACTGTCTTGTGAAATGGTTTCCTTGACAAAATTT<br>AAGCTGAGCTTAAAAGCAAAAAACAAAAAGTACACAGAAATATTTATTAAAATGTAATACAGTTTATTG<br>AACTTTCTAGGTATGGAGTTTGATGGACAGGGCTGCCTTTAATGAGTGTGAA[...]TATTTTATTTTATTTT<br>ATTTTTTTGAGACAGAGTCTCACCCTGTTGCCCAGGCTGGAGTGCAATGGTGCGATCTTGGCTCACTGCA<br>ACCTCCACCTCCCAGTTCAAGTGATTCCCCTGCCTCAGCCTCGCGAGTAGCTAGGACTACAGGCGCATGT<br>CACCAAGCCCGGCTAATTTTTGTATTTTTAGTAGAAACGGGGTTTCACCATGTTGGCCAGGATGATCCTC<br>AATCTCCTGACCTCGTGATCCACCCGCCTTGGCCTCCCAAAGTGCGGGATTACAAGCGTGAACCACCCTG<br>CCCAGCCAGAAACTAGATTTTCTTTATGCCTCCACCCCTTCTTATTCATTTACTTTACAATACCAAAATAAC<br>AAATTGCATAGGAGTGTGGGATGTGGTTTCTGCCTTCTAGAGAGAGATCAGAATTAAACTAGTACTATG<br>AAATGTCCTTTTGAATGTTAGGTCAAGAAATCCATGTACAGAGTCGTGCACTTCATTGGCTGTAACTGTC<br>AAACTTGCCTTGTATTAGAAGTTAAATCCCATCGTAAATACATCACGAGGCCAGCTGTGTGATTTCTGAG<br>ACCTAGATGAGAGTCCATTTACTTCAGCCTTTGATTCAGTAAATGTTAAGCAGCAGAAGACTTCGAATGG<br>TTGAAATTCATTGTTACAGGATTTAACTCATAAACCAATGAGAGATTTTTTTTTTCTCTGCAATGGTTGCT<br>AAGTCTATTCCATGTTAACACTGTGGAAATAAATATGAAGATGTGGACATTGC
**GCH1**	CTTCATTCAGTGTGTGTCTGCAGAACCATAGCTTCCACGCACCTGAACGAGCACAGAATGAACTGACGG<br>TGGAAGACATTATGAGCTGTGTCCAACGTTTTAACCAAAGCGTATCGTACCAACGATCTGTGAAAATGC<br>ACTGGAAGCTTCTGGTCCCGGTTTCCTTTGTGGTCTATGTGGGTCTTGTCCTCATTGTAACTCCGTATAGA<br>TGGTATAGGTATTTTAATCCTGGAAGCTGTTGCCTTATTAATGATTATCTTAAAATTTCCTCCATTGGGGC<br>AGCGTGGGCCAAATTAAAACAAACAAAACCGCAACTCCTCCACAGAAACACAAACACAGTTATTCCATG<br>AAGTTTAGTATTTGGTTGACATAGTGCTCTTCAAATTCATCCCATTACCCTAAAAGTAATAACTTTGATGC<br>TTGCTTTAACTTTAGTCCCATCTCTGCCACTTTGATGCTATTTGGGTTATGATGGGGCAAGATGGCAGAG<br>GTATTGGGTTTTTTTGTTTTTTTCCATTCCTCTCTACTTCTGTTTCCTAGCTTTTTCTTTCTGGAGTTTAAGT<br>ACAGTGATGGTTGGCTTGAGTACCTTTTTAAATCTAGCCCAGTATAAACATTAGCCTGCTTAATATTTAGA<br>CATTTATAGGTAGAATTCTGAGCACTCAACTCATGTTTGGCATTTTAAAGTAAAAACAAGTGTGACTTCG<br>AGGACCAAAGAAATTGTCAGCTATACATTTATCTTTATGAACTCATTTATATTCCTTTTTAATGACTCGTT<br>GTTCTAACATTTCCTAGAAGTGTTCTTATAAAGGTCTAATGTATCCACAGGCTGTTGTCTTATTAGTAAAT<br>GCAAAGTAATGACTTTGTCTGTTTTACTCTAGTCTTTAGTACTTCAAAATTACCTTTTCATATCCATGATCT<br>TGAGTCCATTTGGGGGATTTTTAAGAATTTGATGTATTTCAATACACTGTTCAAAATTAAATTGTTTAATT<br>TTATGTATGAGTATGTATGTTCCTGAAGTTGGTCCTATT
**GIN1**	TATTCATTAAAAACGTTGTATAGAAGAAGTATCTTGACACATTCTTAATGACTATATTCTGAAGGTTTATT<br>TTATAGTTCTTATCTAAAATTATGCCACATTTGAATATCAGTATACGTACATATGAAGGCAAAGGAAGCA<br>TGTAATATATAGAATTCACTTTTTCTTCTAAAAAGTGAACAGGCAAAGCACCCCTACTTCTTGGGAAATA<br>AATTCCAGCCCAAATCTCTCAAACCTTTGTATGGTTTTGGCACCGTTTCTAAGTTTTAAATTTTCTGAAATT<br>CTCTACCATTTAAACAGTAAGTTTGAACTACCTTGAATGGAATTGATAAATACTGAATCAACTGTAACAA<br>TGAATGAAATGAAGCTTCTTTCTGTTTCATTAGAACTTCTAAAAACAAGATTGTATGAAAAAATATAGGG<br>TTATTTAATAGGCTTTCACAGAATATTGGTAATTTCAAATTATCTAATTGGTTAGATAGGGTCAGGATTA<br>GGGTCTGAAACTATGTATTGGTTGGGAAACAATTTGTTTATTTTCATACTATATATTAAAGATAAAACCAT<br>CGACCAGGCATGGTGGCCCACACCTGTAATCCCAGCGCTTTGGGAAGCCAAGGCAGGATGATCACTTG<br>ACCCCAGGAGTTCGAGACCAGCCTGGGCAACACAGGGAGACCCTATCTCTACAGAAAAATGTTTAAAAA<br>TTAGCCAGGTGTGGTGGCACATGCCTGCGGTCCCAGCT[...]AAACTATGTAAGCCATGTACAACACAAGT<br>TAAGGCATGCTAAATTGCAGAAGACAGGTGTAGATAATGTACTTAGGGAGGCAAAAAGAGAGAAGTTG<br>TTTTTACATAGGAAATTTGTGTTTATCCTTATGGAAGAAGGATCATTTAAGCTTGACCTTGAAGAATGTA<br>TAGGATTTGGTAAATGGATACAAAAGAATTAGGGGAGAAGTAATACACAGAAATGGAAAAGTATTGTA<br>ATGATTCATAAAAAGGCAGGGCTTCTTGTTGTCTACACTGCCTCGGGTATAAAGTGAAGAAGCAAGATA<br>ATGAGGGATAAAGCTGAAAAGGTAAGTAGGAAACCTTATGGAGAGCTTTGAATACTATTAAGGATTGA<br>TATGTGCCCCTCCCAAAATTTGTGTTGAAGTCTTAACCCCCACCACCTCAAAATGTGACCTTATTTGGAAA<br>TGAGGTTGTTGCAAGTGTAATTAGTTGAGATCATATTTCAATAGGGTGGGCTCTAATACAGTATTACTGG<br>TGTTCTGATAAAAGGGGAAAATTTGAACACAGACTCAAGGAGAATGCCATGTGAAGATAAAGGCAGAG<br>TTTAAGATTATGTTTCTATATGCCAAGAAATGACAGAGATTGCCAGAAAACTACCAGAAGTTAGGGGAG<br>AGGCATTGAATACATTTTTCCTCATTCCTCAGAAGACCAACCCTGTCAATACCTTATCTCAGACTTTTGCCT<br>CCAGAACTGTGAGACAATAAAATTCTATTGTTTCAGCC
**IFIT2**	GAATAGAGATGTGGTGCCCACTAGGCTACTGCTGAAAGGGAGCTGAAATTCCTCCACCAAGTTGGTATT<br>CAAAATATGTAATGACTGGTATGGCAAAAGATTGGACTAAGACACTGGCCATACCACTGGACAGGGTTA<br>TGTTAACACCTGAATTGCTGGGTCTTGAGAGAGCCCAAGGAGTTCTGGGAGAGGGACCAGATTGGGGG<br>GTAGGTCCACGGGCTTGGTGATAGAATTATTTCTCGATTGACTTCTTGAGTGCAATTTGAACTGTAACAT<br>TTGCTTAGTCACCTTTAGTGGAGTAATCTACTGGGCTTGTTTCTATATTTATATAAAGCAGCCAAATCCTT<br>CATGTAATATTGAAGTCCATTTTTGCAATGTTGTTCCATACTTGGAGTCATTTTGCATCCCATAGAGGTTA<br>GTCCTGCATAGCCAGTAATGTGCTAAGTTCATCCAAAAGCTGGCGGACCAAAGTCTAAATAGGGCTCAG<br>TATCCCCCATCGCTTATCTCTGCCTCCTTCCTCCTCCTTCCCAGTCTATCATCAACCTTGAGTATTCTACACA<br>ATGTGAATTCAAGTGCCTGATTAATTGAGGTGGCAACATAGTTTGAGACGAGGGCAGAGAACAGGAAG<br>ATACATAGCTAGAAGCGACGGGTACAAAAAGCAATGTGTACAAGAAGACTTTCAGCAAGTATACAGAG

	AGTTCACCTCTACTCTGCCCTCCTCATAGTCATAATGTAGCAAGTAAAGAATGAGAATG[...]TGAGAAAT<br>CACAAAATTCACGGTATGCTTGGAACGATTGAGATTTTCTAGGTAGATGCTGAATAGCCTAGACATCAA<br>AGTTGGTGTGAACCAAAATAGAGTCAGCTGACCCAGCATCAGCCACACTCTGGGTTGGAAAATGTTTGC<br>CTGTTGGAATTAATTTAAGCTTAAGTATATATCAACATTATTTTATTGTGCAATTAAAACAATACAAATTC<br>ATGGTTTTTTAAAGTTAAAAATTCTAACCACTGTAACAACAGTTTTTGTGTTATTTTCTGTATTAAACATCT<br>TGTTGCACGCATTTGAGGTCATCAGGGTGCAAAATTTGTATTCCTGAAAATGTCATATATTTTCATTAATA<br>AATAACCTAAATATGATAAAACATAAAGCAGTGTTCTGGTTCATCTGGAATTTTGCTGTACTTTAAATCTT<br>TCAGACTCAGCTACTGATAAATGAAACGTTACACAGGTGTGAACCAAATCCAAATAACCTCGACTGGTCT<br>ACTATCATAATCACCTGAACAGAACAAAACTTTTTCCTCAGCTTTAAGAGTCCAGGGCTTCGGATAACAG<br>CTGCCATCTGCCACCTGCTACCATTGACCTACGTGAACACAGACATTCTGTCTCCACCTTGATGATGGGT<br>GGGCTGCTCCCCTTTTCTTTGTTAAATTTTGTGCTTTCATCACATTTTCTCTATTCTGACCTCTGTTATGAG<br>AAATAAACGTCACTGATTCCA
**ING4**	TAAGGGCCTTGGATTCCAACACAGTTTCTTCCACATCCCCTGACTTGGGCTAGTGGGCAGAGGAATGCC<br>TGTGCTGGGGCCAGGGGTTCAGGGAGGAGTGGATGGCACAGTGCTGTCATCCCTTCTCCTCCCCTCTCC<br>CCACTCCCGGTGCTGAGGCTGCATCAGACCCTGGTAGGGAGGGGTGCCGCAGCCACTAACGGTATGTG<br>CTCTCCTTCAGCCCTCTCCCTTCGGAGGGACGTGGTCTTGCCCACTGTCCTTTTGCCTCCATGCTGAGGTC<br>GGTGCTGTATTTCAGAGGGAGGGTCCTTTTCATTCTCCTTGCTTTGTATTTAAGGACTGGGGCATAGCAT<br>GGGGGCAGTCCCCCAGACCTCTTCATTCCCCCTCCTGTGGTGAGGGCTAGGTGTGATCAACACTTTTCTT<br>CTCCATTCCCTTCCTGCTTTTTTCATGGTGGGGGATCCACCAGGTCATCTAGGCTCTGGCCCTAGTTGAAG<br>GGGCACCCCTTCCTCTGTGCCAAGAGGATTCATCCTGGGAGAGGGGGCAAGGTGGAATGCAGATAACT<br>CACATGTAAAAGGAACTTGGGTAGGTAAATAAAAGCTATACATGTTGGCCTGCTGTGTTTATTGTAGAG<br>ACACTGTTTTAGTAAACATGCTGAGCATTC
**IRF3**	CCCTCGCTCCTCATGGTGTGCCTCCAACCCCCCTGTTCCCCACCACCTCAACCAATAAACTGGTTCCTGCT<br>ATGATC
**MAP1LC3B**	CCCATGTCGGGAAGGATGTGTGCTCTGACAGACGTGTCAGATGCTGGCAGAAGGGATTGGTTTTCTCCT<br>GTGTATACAGTGGAGGAGTCTGAGAAGCAGGGATGCCTGGGGTGATCAGCTCCAACCAGTGGCAGCA<br>GAGTGGTGGCTCTTCCTAGTTTAGTTTTTGTGCTCCGTGTTCTTGTGTCCTTCTAAGAAAAATGTGGGCT<br>GCTCTTGAAAGTTATATAATTATCATTTTTTTTTTTTTGAAACAGGGTCTTGCTCTATTGCCTAGGCTGGA<br>GCACAGTGGCATGATCTTGGCTCACTGCAACCTCCGCCTCCTGGGTTCAAGTGATTCTTCTGCCTCCACC<br>CCCCGAGTGGCTGGTATTACAAGCACATGCCACCACACCAGGCTAATTTTTGTATTTTTAGTAGAGACGG<br>GGTTTCACCATGTTGGCCAGGCTGGTCTCGAACTCCTGACCTCAGGTGATCCACCCACCTCGGCCTCCCA<br>AAGTGCTGGGATTACAGGCGTGAGCCACTGCATCCAGCCATATAACCGTATTCTAAATAAGAAATGGTT<br>GGCTTGTGTGATGGTTTTGTGTAATGAGCTAGAGATAATATTTTAAGTGTCTTCTGTGGTATATGTGGGA<br>GGGCCATTAAGGAGTGGGTTTCACTCCCTGCATGTGGGCAGGTGTCC
**MFN1**	AATAGAGATTGCTTTGGTGACCATGATAGGAGGAAACGAAACTTGTAAGATTGGAACAGTTGTTATTTT<br>TATGAAATTACTTTAAATATGAATTGTACTAACTGTACCTAAATAGCAAAGCCCTGTGTAGATTCTGGTA<br>ATGATCTGTCTCAGGGTATGTGTATTTTTGAAGAGTGTTATGTCCTTAGTTTTAATTTTGAGTAAAGAAA<br>AGGCTAAAATCATGAATTAGTTACAAGCAACAGTACCAACTTATGTGACCCCTGAGGGGTGGGGCTGTG<br>AGCTCTTAATTTGTTTTTGATTCTGAAAAACTCTGCTTCCTGGCATCCAGGAGTTAGAGATTGAGCCTTTC<br>ATCTTCTTTCTCAAAACTAGTTTTTGATGCTTTCTTTCATGGGAATAGTCACTTTTTTATTTAGTAAATCGC<br>ATTGCTGGAACCACCAAGGAGTGTGGAATGTCCTTGAGTGTATTATTTATGCAAGTCACAGTCACGTTG<br>CCATCATGGCAGCTATGTGAAACACTAATAAATGTGTTTTTACTTTTTATTCCCGTTAAAACTGATGTAAA<br>ACAGGATAAAGGCTTGTTATAGTCACTTATAAGTATCTGGGTCTAAGTAATTTCCTTAGATGTTTCTAAA<br>GAAACATTTTCAGCTTTGCTCCCATTATGATTCCAATAAGGAACGCTTTCCTAGTGCAATTTTAGGAGTAA<br>AGTTTGAAGAGATAAAAATAGCCAAAGATAGGAGACGTCTGAATTTTGAATGATAAACAGTGATGTTTT<br>AAAAAAGCTGTTGTTCTTCAGGAGGCATTTGCCTAGGATATTGCTGGATTATACCCCATTGGAGGCTTTT<br>AATTTTATTTGTATGAATTTTCCAGGATTTCATTAAAAATTATTATTGTATTTTTTACCTTAATGAAAGATT<br>TTGGGTTCAAATATCTTTCTATATTAAAAGCTGATTGAGTCTGTACATATGTAAATTATGCCTAGTGGAG<br>GTTCTGTTGACTTTCTTCCCCACTGTGGAAGAGGCCAGTTTTGCCTCCATTTGCACATTCATTTCAGTTATT<br>TCTGATCCATAAATATAACATTTACAAAATTCTTCCTTGAGCTGGTGGAAATGCCTCACCAGTTTC
**MLLT10**	CAAGACTTAGTGATAAAACTGGGCCTGTAGCTCAAGAGAAAAGTTGACACCTGAGAAACATCTAGAAAT<br>TGCCTATCCTGCTGTTCTAGCACTTCATCTGGCTGCCTTTGCAGTCCTTTTACTACAGCTATGAAGAAACG<br>CAACAAGAAACTCAATGCACAACAAAGGATTAATTGCTGCAAGGACATTCTTGTAAGGCTTTGATTAGTT<br>TTCTTGTTGCTTTGTTGCACTGAAATGGAATTCCCATGCCCCTACCCCTTACCCCAGTTTTTTGAACATGG<br>AAAGAAAATTTAATAACTTTTTAAAGTGACATAATTTACATGCAATATGTTTATCAACTCAAGAATTTAAT<br>ATAGTTGGTACACAACTAGTTTTGTTTATAAATTGGAGATGCAAATAGCAAAACTAAATACTTGCTCCAT<br>TTACAAACTACTTGATTTTATTGTACAAGTTGAAATATGCTCTTTTGTTTGGGTTACAGTATGCTTGCTCTA<br>AGTCAAATTCCAAGGAACTAATTTCTTCTCCTGGAGTTGCATTGATTCAGTATTACAAATATATAGCACAT<br>CACCTGGGACTTGGCAATCTTTGTTAAAAAAAAAATTTCCTTTCTAATGGGATTTGGCCAATTTTGGTAAT<br>GAAGTTAGGATGGTAATGTCTGCATCTGCTAAAGGTAATTTTCTTTTGAGAATTGCTTTCTTTAGTGTTAA<br>GACCTACTCATATTTTGAAGAAATCTTGAGTTAAGTGAGTTCTGAGGCTGCTGGGGGAACCAGATCAAT<br>TCAAAGCTAAATACTTCTTTCAGAAAGGGGCCACTGTGGAAAGTGCTGGTGGGGTTTGCCCTTGATCAA<br>GTGCCTCCATTGTGCTGCAAGGGCTGTAGACAGCAGGGTGGGACAGTCAGTCCTCCGAGCAGCAGGAA

	TCATCCCGTCACTGCAGCCTTCCCATGCTTCCGCCTTTATTCAGAACTTTCTGTGCCACTGTAGATAGCTC<br>AGGCAAAACTATTACCTGGGTATTTATCCACTAATGAGTCACAAGAAAAGGAGTGGATTTGGTAAGAAT<br>AGAGATTTGTTTTATTTAAACCACTTCCCATTACTGACCATTAAAAGCTCACCACTAGAGTTCCTGAAACA<br>GGTGAAACCTGTATGACAGCCCTTCCACTTTGGGGAGCCACGCTTTTGATGTGACAGTACCGCAGAGTG<br>ATTCCCCCACTGAGGATGTCATCATCAAACTCTTCTTTGGTGTGTGAATTATTAGTGGAAAAGACCCAGC<br>TGTAATTAGACCTCCACTGTGTACTTAGCTGGAAGAACATGTTAATTCTGCAATATGTTTCTTGGTTAAAC<br>ATTGCACAGTTCTTACCTCATTTCTGTAAATAAAGTTTTGTGAATCTGTTTTGTATTGTGACAAATTCATAA<br>GATAACATTGATATTTTGATTTGTAATATTTCTAATTGGTAGATTTAATTGAAAAGTAAAATTAATTTATT<br>TTTATATGTTCAGGGGAATTTTAAAGTCAAATCTTTTGTAGATAATTTAAAAAATCAGTGTGGTTTATTTT<br>ACTTATTTAACCCACTGGTTGTTATTCTGTAACAGTTTGTATAAATGGTAAATTTTGAATGTGTTGTTATTT<br>TACCTAGATGTAAAATTCCACATGTATTAAAATGTACAAAATGT
**MxA**	CCCACACTCTGTCCAGCCCCGTAGACGTGCACGCACACTGTCTGCCCCCGTTCCCGGGTAGCCACTGGAC<br>TGACGACTTGAGTGCTCAGTAGTCAGACTGGATAGTCCGTCTCTGCTTATCCGTTAGCCGTGGTGATTTA<br>GCAGGAAGCTGTGAGAGCAGTTTGGTTTCTAGCATGAAGACAGAGCCCCACCCTCAGATGCACATGAG<br>CTGGCGGGATTGAAGGATGCTGTCTTCGTACTGGGAAAGGGATTTTCAGCCCTCAGAATCGCTCCACCT<br>TGCAGCTCTCCCCTTCTCTGTATTCCTAGAAACTGACACATGCTGAACATCACAGCTTATTTCCTCATTTTT<br>ATAATGTCCCTTCACAAACCCAGTGTTTTAGGAGCATGAGTGCCGTGTGTGTGCGTCCTGTCGGAGCCCT<br>GTCTCCTCTCTCTGTAATAAACTCATTTCTAGCAGACA
**NOD1**	AGGATGCTTTCCTGTTCATGGGGTTTTTGCCCTGGAGCCTCAGCAGCAAATGCCACTCTGGGCAGTCTTT<br>TGTGTCAGTGTCTTAAAGGGGCCTGCGCAGGCGGGACTATCAGGAGTCCACTGCCTCCATGATGCAAGC<br>CAGCTTCCTGTGCAGAAGGTCTGGTCGGCAAACTCCCTAAGTACCCGCTACAATTCTGCAGAAAAAGAA<br>TGTGTCTTGCGAGCTGTTGTAGTTACAGTAAATACACTGTGAAGAGACTTTATTGCCTATTATAATTATTT<br>TTATCTGAAGCTAGAGGAATAAAGCTGTGAGCAAACAGAGGAGGCCAGCCTCACCTCATTCCAACACCT<br>GCCATAGGGACCAACGGGAGCGAGTTGGTCACCGCTCTTTTCATTGAAGAGTTGAGGATGTGGCACAA<br>AGTTGGTGCCAAGCTTCTTGAATAAAACGTGTTTGATGGATTAGTATTATACCTGAAATATTTTCTTCCTT<br>CTCAGCACTTTCCCATGTATTGATACTGGTCCCACTTCACAGCTGGAGACACCGGAGTATGTGCAGTGTG<br>GGATTTGACTCCTCCAAGGTTTTGTGGAAAGTTAATGTCAAGGAAAGGATGCACCACGGGCTTTTAATT<br>TTAATCCTGGAGTCTCACTGTCTGCTGGCAAAGATAGAGAATGCCCTCAGCTCTTAGCTGGTCTAAGAAT<br>GACGATGCCTTCAAAATGCTGCTTCCACTCAGGGCTTCTCCTCTGCTAGGCTACCCTCCTCTAGAAGGCT<br>GAGTACCATGGGCTACAGTGTCTGGCCTTGGGAAGAAGTGATTCTGTCCCTCCAAAGAAATAGGGCATG<br>GCTTGCCCCTGTGGCCCTGGCATCCAAATGGCTGCTTTTGTCTCCCTTACCTCGTGAAGAGGGGAAGTCT<br>CTTCCTGCCTCCCAAGCAGCTGAAGGGTGACTAAACGGGCGCCAAGACTCAGGGGATCGGCTGGGAAC<br>TGGGCCAGCAGAGCATGTTGGACACCCCCCACCATGGTGGGCTTGTGGTGGCTGCTCCATGAGGGTGG<br>GGGTGATACTACTAGATCACTTGTCCTCTTGCCAGCTCATTTGTTAATAAAATACTGAAAACACTCTT
**PCSK7**	CCTCAGGGCCTGACAGTGTGGGACAGGCTCTTCTTTCCCAAAATTAGGGAGCTCTTGACAGAAAGCAGT<br>TCTGATGCTTACATCTGGAATCTGAGGCATCCTCTGACTCCACTCAAAGAGGGTGAGGGCCTTCTTAAGA<br>TACAAATGGTGGAGGATTGCTGCCAGAGAAGTCTGGTCAGAGCCACAGGGTCTGCCTCCAGCCAAACG<br>GGAGCTTTTGGTGAGAAGGTGTTGGACAGGGGATTGGCGCCCCCCTTTGGTTTGGCCTCCATCCTCATC<br>TCTCTTGGGCCAAGCCAGCTGCCTAGGTCCCCCAAGCATGGGGGACCCCTTCCCACATATAAGTTGAGA<br>AGGTGCCTGCCATAGCCAGGAGCGCATCTCAATGGAAACATCACTGGGGTCACTTGGGAAGAGGACTT<br>CGGGGTAGAGGCTGGGAGGAGCCCCTGGACATGCCTGTCCTGAAAGCGGCTGCCTCCATTATCCATTCC<br>CAAGATGCCTGATCAGAAACCAACCATGAATGAACCCCTGGCTCCTTCACCACCCCCACGATTGGTATGA<br>TGCTGCCGGCACAGCTGGGATACACACGGCTCCCCCAGGCCTGAGCTGCTTCACTAGGGAATCCTGCGG<br>CAGGACTGCAGAGCAGATGGCAGATGCACATGTTGGAGGAGAGAGCCTTGGGAGCCACTGCCACTCCA<br>GTCCTGCCACCACCCTGTCTTCCTCTGCAAGTGCTCAGGGAAATGGCCTTCCCGCCGGAGGCCAGCTATC<br>TGCCTGACAGGCTGTGACTCTTCTCTCAACCTTGGCCTTCTCCCCTCTTCTGAGCTAGTTGGTTGAATTTTT<br>TTTAATGCTTAAGATTTGTTTTTCTCTTTTCACAGCAACATTTTCTTGAATTTTTTTCTGCACAGCTTTTCCA<br>AAATAAAAACCTTCCAA
**PTPN7**	[...]AACATGTGGGGACTACTCAGAGGCAAAGAGGAGCTGCTCCTGCCTCCATGGTTGCTGGCCACTCCC<br>ACCAACTACTCTTAGGGAGGCTAAGCAGTCTCTGTTTTGCTTCCATGGCTCAAATAATACCCTGGGTATG<br>CAGGACCCACTATACCTTGCATTTGCTGAGTACACCTAGAGAGCTTGGCTGTTTCCAAAAACAATCAGGG<br>TCATAACCATCCATGCAGACATGGAGGCTCGGCTGAACCAGGACTCCTCACTGTCTACCTGAGAGAATG<br>AGCACCCCTCATCCATCTCAGCATCAACACAATTTCCAGGGGACCTCAGGTCTACCTCAGGACTGAACGC<br>CACACCTCAGGATTCCTCCTCCTTGAATCTGAGACTGGCTGCCCATTCTGAGATGGGGATGAAGGTAAG<br>ATGCCGCATCACCAGGCACGCCGCCCCTGACAGCTGCCTTGATACCAGCTCTCTGTGGAAACCCCCGAG<br>GAGTTGGATCTGGAGAACAGCTGGGCCTCCTCACTCAGGACTTCTCTCCTGAAGAACACGCAGTGCTAA<br>AACTGAGGATGATTTCCCTAATGCTTCTGCTTGGCCTTATGGAGGAGCTGCTCCTTCCTTACAGCCTTGG<br>GGATGGACTTGCCCACACCTCCACCTCCCCTGAGCCCTGTGAGAGGCACGACTGTCTATGCCAATGAGG<br>CTCGGTGGGGGGCTCTCAAGTGCCTGATCCTGCCCTGGGCTCAGAGCCAGCCCAGAGGGAAGCAACTG<br>CACAGCCCCACAGGCCCTCCCTGGCACTGTCCCCCCAACCCCATCTCAGAGCTCAGAGGGTACAAGCTCC<br>AGAACAGTAACCAAGTGGGAAAATAAAGACTTCTTGGATGACTGACTC
**RAB1B**	AGGGGCACATGGAGTGGGACAGGAGGGGGCACCTTCTCCAAATGATGTCCCTGGAGGGGACAGGAGG<br>TGCCTCCCTCTTCCTCTCCTGGGGCATTTGAGTCTGTGGCTTTGGGGTGTCCTGGGCTCCCCATCTCCCTC

	TGGCCCATCTGCCTGCTGCCCCGAGCCGCTATTCTGTCAGGGTCCCTAAGGGAGGACACTCAGGACCTG<br>TGGCCAGGCAGGGCGGGGGCCTGCTCTGCTGCTGCCTCTAGGTGACTTTCCAAGATGCCCCCCCTACAC<br>ACCTTTCTTTGGAACTAGGGCTCTTCTGTCGGTCTCCCTCCCACCCCCATGTGTGCTGCACTGGGTTTCTC<br>TCCTTCCCCTTCTTCCTGCTGTCCTTCTGCCCAAGAGCTGAGGGTCTCCCCGGCCTCTACTGCCCTGGCTG<br>CAGTCAGTGCCCAGGGCCAGGAGTGTGACCAGGGGATCCAGGGCCCTGGGCTGGACCTCAGGACAGC<br>CATGGAGGCCACAGGGGCCCAGCAGCCCACCCTTTCCTCTCCCCACTGCCTCCTCTCCCCTCCCACACTCC<br>CAGCTCCAGCCGTCCAGCTGCGGTGGGGTCTGAGTATATCGAGGGCGGCTGGGCGGGTAGCGGCGCT<br>GGGCCTGTGTCTTGAGCCTGGAGAGAGTCTGCTCCTGCCGCCCTCTGCCCTGCCAGAGACAGACCCACG<br>CGCTGCCTGCCCACCGTGCCCCTTAGTCCCCATGTCAGGCGGAGGCGGAAGGCCCACCGTGCCAGAGG<br>CTGGGCACCAGCCTTAACCCTCTCACTGCTAACACCTCCTCCCTTTCCCCGAGGCAGCACACCTGGCTCAC<br>TCTCCCCTCGGTCTCTGGAGCCCACCAGGGAAGGCCCTCATCCCCTGCCGCTACTTCTCTGGGGAATGTG<br>GGTTCCATCCAGGGTTGGGGGCCTCTCTGCCCACCCACTCTGCACCCAGGATCCTAGTCCCCTGCCCTCT<br>GGTACAGCTGCTTCCTGCAGGAAAGCAAGTCTTTGGTCTCCCTGAGAAGCCATGTCCCTCGTGCTGTCTC<br>TTGCCTGTCCCACCTGTGCCCTGCCCTCCAGCTTGTATTTAAGTCCCTGGGCTGCCCCTTGGGGTGCCCCC<br>CGCTCCCAGGTTCCCCTCTGGTGTCATGTCAGGCATTTTGCAAGGAAAAGCCACTTGGGGAAAGATGGA<br>AAAGGACAAAAAAATTAATAAATTTCCATTGGCCCTCGGGTGAGCTGAGGGTTT
**RARB**	ACATTTTCTAGCTACTTCAAACATTCCCCAGTACCTTCAGTTCCAGGATTTAAAATGCAAGAAAAAACATT<br>TTTACTGCTGCTTAGTTTTTGGACTGAAAAGATATTAAAACTCAAGAAGGACCAAGAAGTTTTCATATGT<br>ATCAATATATATACTCCTCACTGTGTAACTTACCTAGAAATACAAACTTTTCCAATTTTAAAAAATCAGCC<br>ATTTCATGCAACCAGAAACTAGTTAAAAGCTTCTATTTTCCTCTTTGAACACTCAAGATTGCATGGCAAAG<br>ACCCAGTCAAAATGATTTACCCCTGGTTAAGTTTCTGAAGACTTTGTACATACAGAAGTATGGCTCTGTT<br>CTTTCTATACTGTATGTTTGGTGCTTTCCTTTTGTCTTGCATACTCAAAATAACCATGACACCAAGGTTATG<br>AAATAGACTACTGTACACGTCTACCTAGGTTCAAAAAGATAACTGTCTTGCTTTCATGGAATAGTCAAGA<br>CATCAAGGTAAGGAAACAGGACTATTGACAGGACTATTGTACAGTATGACAAGATAAGGCTGAAGATA<br>TTCTACTTTAGTTAGTATGGAAGCTTGTCTTTGCTCTTTCTGATGCTCTCAAACTGCATCTTTTATTTCATG<br>TTGCCCAGTAAAAGTATACAAATTCCCTGCACTAGCAGAAGAGAATTCTGTATCAGTGTAACTGCCAGTT<br>CAGTTAATCAAATGTCATTTGTTCAATTGTTAATGTCACTTTAAATTAAAAGTGGTTTATTACTTGTTTAAT<br>GACATAACTACACAGTTAGTTAAAAAAAATTTTTTTACAGTAATGATAGCCTCCAAGGCAGAAACACTTT<br>TCAGTGTTAAGTTTTTGTTTACTTGTTCACAAGCCATTAGGGAAATTTCATGGGATAATTAGCAGGCTGG<br>TCTACCACCTGGACCATGTAACTCTAGTGTCCTTCCTGATTCATGCCTGATATTGGGATTTTTTTTCCAGC<br>CTTCTTGATGCCAAGGGGCTAATTAATATTAACAACTCCCAAAGAAACAGGCATAGAATCTGCCTCCTTT<br>GACCTTGTTCAATCACTATGAAGCAGAGTGAAAGCTGTGGTAGAGTGGTTAACAGATACAAGTGTCAGT<br>TTCTTAGTTCTCATTTAAGCACTAGTGGAATTTTTTTTTTTTGATATATTAGCAAGTCTGTGATGTACTTTC<br>ACTGGCTCTGTTTGTACATTGAGATTGTTTGTTTAACAATGCTTTCTATGTTCATATACTGTTTACCTTTTT<br>CCATGGAGTCTCCTGGCAAAGAATAAAATATA
**S100B**	ATTAGAAAGCAGCCAAACCTTTCCTGTAACAGAGACGGTCATGCAAGAAAGCAGACAGCAAGGGCTTG<br>CAGCCTAGTAGGAGCTGAGCTTTCCAGCCGTGTTGTAGCTAATTAGGAAGCTTGATTTGCTTTGTGATTG<br>AAAAATTGAAAACCTCTTTCCAAAGGCTGTTTTAACGGCCTGCATCATTCTTTCTGCTATATTAGGCCTGT<br>GTGTAAGCTGACTGGCCCCAGGGACTCTTGTTAACAGTAACTTAGGAGTCAGGTCTCAGTGATAAAGCG<br>TGCACCGTGCAGCCCGCCATGGCCGTGTAGACCCTAACCCGGAGGGAACCCTGACTACAGAAATTACCC<br>CGGGGCACCCTTAAAACTTCCACTACCTTTAAAAAACAAAGCCTTATCCAGCATTATTTGAAAACACTGC<br>TGTTCTTTAAATGCGTTCCTCATCCATGCAGATAACAGCTGGTTGGCCGGTGTGGCCCTGCAAGGGCGT<br>GGTGGCTTCGGCCTGCTTCCCGGGATGCGCCTGATCACCAGGTGAACGCTCAGCGCTGGCAGCGCTCCT<br>GGAAAAAGCAACTCCATCAGAACTCGCAATCCGAGCCAGCTCTGGGGGCTCCAGCGTGGCCTCCGTGA<br>CCCATGCGATTCAAGTCGCGGCTGCAGGATCCTTGCCTCCAACGTGCCTCCAGCACATGCGGCTTCCGA<br>GGGCACTACCGGGGGCTCTGAGCCACCGCGAGGGCCTGCGTTCAATAAAAAG
**SIRPB1**	TGACCCTCAGTCTCTGCTGCCTCCTTCCTTTCCTGAGAGGCTCAGCTTGAGAGAATGAGCCAGTGAGAAG<br>CTTCTCTAGACTTGGCTCCAAACATCTCCCCTCCCAAGACATCTGCCTGCCCACAGGCTCCTGTTGCTCCT<br>TCACACAGACCTGGATGCCCCAGAGCAAGGTCTTCATTCATGGTCCTGAGCAGGGGCCATGGGATTGG<br>GCTCTGGGCACTGACTTAACGGCACCTCCCTAGAAGGCGAGAAACATGCCAAATCTAAACACACCAGGA<br>CTCCCATCCATCGCCTTGAGACTGACCGTAAACCACAGACGCTCTCCAGGTTCTCAAGAGTTATCCTGCC<br>TTCCAGATTCCTGCCTATCCCAACTCCCCAGCCTTGTTGAGGTTCTCTATTGCCTCTTGAATACAAATGCA<br>CTCCCAAAGTGGTTTTAAGAAAATAAAAAGATTATCCTTCCTCTTTGTCTAAGGGTCCTACTCCTCACACT<br>CAGAAACAAAGAAGCTCTCCCTGCTCTCTCTGTGATGGGAACACTAGTTCTAGCATCCTGCAGGCTTCCC<br>CGCCCTGCTGCTCATGGCGGAAATGCTGCCGCAGTCCAAAAGCCTCCTTGACTCCCGAATCCTCCCTGAT<br>GTCTCTTTACACCCCATATTCAATTCTGCCTTCAAAATACTTATGTCCACTCGTGAGATGGTAAGTGTTTA<br>AAAATGGACTCTCTTAAAGGAAAAAAAAAAGCTGATTTGTTGTACTTGACAATTTCTGTGGTGTAATCACC<br>CCCAACATGGCCAGTTTCAAGCTACCAGAAGTTTAACAACTGGATTGCAAAATTCCTGAAACTTAACGGT<br>TGGCTTTCAGGGGCCAGTCCAAGCCTGCTGCAGGAGAGCACTGAGTTACATCCCTAACCTGCACACTTCT<br>CACCTCCATCACCCCTCCCTGTCCCGGCCAGCATCATCTCTTCCGGGAACACTTCTGTAGCCTCTGTTGGC<br>TTTCATTTTTCAGCTACAGGGATTTTTGGAAAAGTCAAATTGTATCACTCCTCTGCTTCTTCCCCAACACA<br>GCTGCATGGCTCCCTTCATCCCTCCTTCAAGTCTCTGCTCAATGTCACTTCATTA

**SOCS3**	GGGAAGGGCGCAAAGGGCATGGGTCGGGAGAGGGGACGCAGGCCCCTCTCCTCCGTGGCACATGGCA<br>CAAGCACAAGAAGCCAACCAGGAGAGAGTCCTGTAGCTCTGGGGGGAAAGAGGGCGGACAGGCCCCT<br>CCCTCTGCCCTCTCCCTGCAGAATGTGGCAGGCGGACCTGGAATGTGTTGGAGGGAAGGGGGAGTACC<br>ACCTGAGTCTCCAGCTTCTCCGGAGGAGCCAGCTGTCCTGGTGGGACGATAGCAACCACAAGTGGATTC<br>TCCTTCAATTCCTCAGCTTCCCCTCTGCCTCCAAACAGGGGACACTTCGGGAATGCTGAACTAATGAGAA<br>CTGCCAGGGAATCTTCAAACTTTCCAACGGAACTTGTTTGCTCTTTGATTTGGTTTAAACCTGAGCTGGTT<br>GTGGAGCCTGGGAAAGGTGGAAGAGAGAGAGGTCCTGAGGGCCCCAGGGCTGCGGGCTGGCGAAGG<br>AAATGGTCACACCCCCCGCCCACCCCAGGCGAGGATCCTGGTGACATGCTCCTCTCCCTGGCTCCGGGG<br>AGAAGGGCTTGGGGTGACCTGAAGGGAACCATCCTGGTGCCCCACATCCTCTCCTCCGGGACAGTCACC<br>GAAAACACAGGTTCCAAAGTCTACCTGGTGCCTGAGAGCCCAGGGCCCTTCCTCCGTTTTAAGGGGGAA<br>GCAACATTTGGAGGGGACGGATGGGCTGGTCAGCTGGTCTCCTTTTCCTACTCATACTATACCTTCCTGT<br>ACCTGGGTGGATGGAGCGGGAGGATGGAGGAGACGGGACATCTTTCACCTCAGGCTCCTGGTAGAGA<br>AGACAGGGGATTCTACTCTGTGCCTCCTGACTATGTCTGGCTAAGAGATTCGCCTTAAATGCTCCCTGTC<br>CCATGGAGAGGGACCCAGCATAGGAAAGCCACATACTCAGCCTGGATGGGTGGAGAGGCTGAGGGAC<br>TCACTGGAGGGCACCAAGCCAGCCCACAGCCAGGGAAGTGGGGAGGGGGGGCGGAAACCCATGCCTC<br>CCAGCTGAACACTGGGAATGTCAGCC
**TGM4**	CTTGTCTGATGCTGTGGAGCCTTAGTTGAGATTTCAGCATTTCCTACCTTGTGCTTAGCTTTCAGATTATG<br>GATGATTAAATTTGATGACTTATATGAGGGCAGATTCAAGAGCCAGCAGGTCAAAAAGGCCAACACAA<br>CCATAAGCAGCCAGACCCACAAGGCCAGGTCCTGTGCTATCACAGGGTCACCTCTTTTACAGTTAGAAA<br>CACCAGCCGAGGCCACAGAATCCCATCCCTTTCCTGAGTCATGGCCTCAAAAATCAGGGCCACCATTGTC<br>TCAATTCAAATCCATAGATTTCGAAGCCACAGAGTCTCTCCCTGGAGCAGCAGACTATGGGCAGCCCAG<br>TGCTGCCACCTGCTGACGACCCTTGAGAAGCTGCCATATCTTCAGGCCATGGGTTCACCAGCCCTGAAG<br>GCACCTGTCAACTGGAGTGCTCTCTCAGCACTGGGATGGGCCTGATAGAAGTGCATTCTCCTCCTATTGC<br>CTCCATTCTCCTCTCTCTATCCCTGAAATCCAGGAAGTCCCTCTCCTGGTGCTCCAAGCAGTTTGAAGCCC<br>AATCTGCAAGGACATTTCTCAAGGGCCATGTGGTTTTGCAGACAACCCTGTCCTCAGGCCYGAACTCACC<br>ATAGAGACCCATGTCAGCAAACGGTGACCAGCAAATCCTCTTCCCTTATTCTAAAGCTGCCCCTTGGGAG<br>ACTCCAGGGAGAAGGCATTGCTTCCTCCCTGGTGTGAACTCTTTCTTTGGTATTCCATCCACTATCCTGGC<br>AACTCAAGGCTGCTTCTGTTAACTGAAGCCTGCTCCTTCTTGTTCTGCCCTCCAGAGATTTGCTCAAATGA<br>TCAATAAGCTTTAAATTAAACT
**TMED2**	AAGCCTCTTCCTGATGATCCCAACTCAGAATTCACTGTTTACCAAACACCTTGGTCATAATAATGTCATTA<br>GTTTCTCCATTTTTATTTTCTGAACTGTACATTCACAACTTATGTTTCTTTGAGATTAATAGATATTGGGGG<br>AAAAACGCCTTTTTAGGAAAATTATAGTGAAAATTTGACAGTTGATTGGCATAATTTCTTGTTTGAATGC<br>TGCCTCCATTATATAGGTCCTTCCAGGAACTCAAACACTGTAAGTGAAATATGGGAGTATAGTTTTTATT<br>ATTTCTTCTTTTCCTTTTGTTTTCATAATATAATGCAGTTTGTTCAGGAAATCAGCACAAAGCCTGATAGTA<br>CTTTACTAAAATGACTGCATTCTTTGGATTCCTTCAGTCTATGGTTCAAGTCACTAAAGATTCATTTTTGTT<br>GAGTCCTTATGAGAAACAGCAGTATGAATCTTGACGGTTTCTGCCCGTCCTAATGGCAGAGCTCTCTGAC<br>TTGGGTGTATGCTGCCAGGCTGGGTACTTTCATACTTTGTTTTCTTGTTTTGCTTTAAAACTACGACTCAG<br>CATACATTTTCCCACATACATTTTTACATTGTACCTTAGGACTCAGTCATCTCCACTTAAATTGATGACACA<br>AGCAGCTAATAACCATTTCTGGGTTTCTGCCTAACCCCCTAATTGTCTGTTAAAGCCAATTCTCTGGGTGT<br>CCCAGTGAGTGGTGGCTTTTTTTCTTTCCACATTGGCACATTCACTTCTCCCACTCTTGGCATGTAAGAAA<br>TAAGCATTTACATAATTGGAAAAATCTGGATTTCTGATGCCAAAGGGTTAAAGCTTCTTGGATTTCATTTC<br>ATTGATATACAGCCACTATTTTATTTTTGATCAGTGGCCTTTGGGCCACTGTTCAGGGTACTGACCATCAG<br>TGTCAGCATTAGGGTTTTGGTTTTTGTTTCTTTTGGGTATTTCTTTTTTGGCACATGTGAATCTTGTTTTGT<br>GTAAAATGAAATTACTTTCTCTTGTTCTCTGATGATGGGTTTAAAATTAAAAGAGCATCCGGTTTTGGTAT<br>GGGGATGATCCAGGATTATGTTGTGACTGATACATATTAGTTACTTGTGCTTTTTTTTTTTTTTTTGGATCT<br>TTGCAAGGGCAAAACTACAAGTAACGAGTTTTATATAATTAATTTAAATTTGTTACAGGTTTTCATGTTCA<br>GGATAAACCATACTTCCACCTTGGGTGAGAACACTTGCAACAGTTTATTAATGAGGTGACTTTCACCTTA<br>GGACAACTGTTGCATGCCAAGTTTTTTGTGTGTGTGAAACACTTCAAAACTGATTTAAAAGATGTAAATT<br>TAAAATTGGTTGTATCTAATATGCCCCAGGTTCGGTAA
**TRPC5**	TCAAGCCCTAATCATCTGTTCTGGAACTCAACAGAAATCTGTAATTTCCTTGCTCTCAGAGGTGACACAG<br>AATATGATTCCCTCACTGCCCCTGCCCCCCAGAAAGGAGAGTGAAACTCTTATTTGCACCATCTTTTATAG<br>CCACATTCTCCATTTTTGTTCTTGTTTTATTATTACTTTCATTATTATTTGCCTTTTTAATACCTATACACACT<br>AAGTCCATCTTACAACCGACTGAAGGGGTGTTGCACCTAATCATCAGAGGTGCAGTTGGGTGCTTAACC<br>TTTTTGCTTTGCTTTTCTCACCCATGCTTCCTTGAAGCCCCAGGTGCCACTTTGCTGGTCTTGTTGCCGCAC<br>AGATTCCTGACCATCCTCAGAATCTCATGCCATTTTCCCGCAGAGCTGAGGGATGTGGTCAGCAGTCCCC<br>ACCAGCTATCAGATAAGGCAAACTACCCTGCCCATCTTTGTTGCCATTCAGTGCTACGCGCTGCCCATGC<br>TGGGTCAATGAACAGCATCAAAGGGAGGCTGAAGAGATTTCAAAGAATTGTCAGCTTGTGGTTTCTGG<br>GTATAAGTGGCTTGTTTCGTTCTTAAATACTACAACTTCTTATTGTGGGGGCTATTGAGTTTTCTTGTTTCT<br>TAAAGATGTGTTAGTTTCCTAATGTGTGCCAATGAAATATTTACCCCATAGTATATGTCATATCTAATAAG<br>TTAGGCGAATTATTTTTCTGTTTGTGTGTTTCAGTTAACTTACTACATTTGAAAATTTGATAGTATCAAAAT<br>[...]TGAAACAACTCTAGGTCTGAGTCCTAGAGATTACATAAATAGTGTGTAGCCCAGAAAAGTAAAATA<br>GGTGTCACGCTGTTTTTTTTAATGTCAACACCAATAGGAATCCATTTAGATTAATGTATGTAATTTCTGAT<br>AAAAGTAAGAGAAATGAGGAACTTTCGAACTGCAAATATGCCTCCAAACTAGCTTTTGATAAGTGTGGT

	TTCTGTTATTTGAATGGATTCCCATCTTTGTCTCTAGCCTGGAGAAGAGCAAAATGGAGGGGGAGGGGC<br>CAGAAATAGGAGGAGGTTTTGAAGAATAAAGCCTCATATCCAGGAAAAAGTGATCAAGTGAAAAAGCT<br>GCATCAAGACCCATTTGGCAAGAGGTTGTCCGAAAATAATTCCTTGATCATATCTGAAGTGGTTATTTAC<br>TCAAATCAATTTCTCCCCTGCTTCGCTAAGACTGTCATAAAATGGCCTTGACATTTAATCACAGGTTACTT<br>TCCAGAAAGGTTTCTAAAGTTACAGATTCAGCTTTTCAAATTGTCAAATTCACTTTGAATAATAATAGAAC<br>CTGGGCTACATTCCTAACTCATATTAACAGCAC
